# Predicate abstraction for hyperliveness verification

**DOI:** 10.1007/s10703-025-00482-5

**Published:** 2025-07-16

**Authors:** Raven Beutner, Bernd Finkbeiner

**Affiliations:** CISPA Helmholtz Center for Information Security, Saarbrücken, Germany

**Keywords:** Hyperproperties, HyperLTL, Infinite-state systems, Software verification, Predicate abstraction, Hyperliveness, Verification, Program reduction

## Abstract

Temporal hyperproperties are system properties that relate multiple execution traces. In finite-state systems, temporal hyperproperties are supported by model-checking algorithms, and tools for general temporal logics like HyperLTL exist. In infinite-state systems, the analysis of temporal hyperproperties has, so far, been limited to *k*-safety properties, i.e., properties that stipulate the absence of a bad interaction between any *k* traces. In this paper, we present an automated method for the verification of $\forall ^k\exists ^l$-safety properties in infinite-state systems. A $\forall ^k\exists ^l$-safety property stipulates that for any *k* traces, there exist *l* traces such that the resulting $k+l$ traces do not interact badly. This combination of universal and existential quantification captures many properties beyond *k*-safety, including hyperliveness properties such as generalized non-interference or program refinement. Our verification method is based on a strategy-based instantiation of existential trace quantification combined with a program reduction, both in the context of a fixed predicate abstraction.

## Introduction

Hyperproperties are system properties that relate multiple execution traces of a system [37]. Such properties are of increasing importance as they naturally occur, e.g., in information-flow control [69], the verification of code optimizations [6], linearizability [63], knowledge [25, 29], and robustness [33, 45]. Consequently, many methods for the automated verification of hyperproperties have been developed [8, 54–56, 77–79]. Almost all previous approaches verify a class of hyperproperties called *k*-safety, i.e., properties that stipulate the absence of a bad interaction between any *k* traces in the system. For example, we can express a simple form of non-interference as a 2-safety property by stating that any *two* traces that agree on the low-security inputs should (globally, i.e., over the entire execution) produce the same observable output [81]. We can express such a requirement formally using a variant of the temporal logic HyperLTL [36] as

$$\begin{aligned} \forall \pi _1. \forall \pi _2 \mathpunct {.}(l_{\pi _1} = l_{\pi _2}) \rightarrow \Box (o_{\pi _1} = o_{\pi _2}). \end{aligned}$$

The formula states that all two traces $\pi _1, \pi _2$ which (initially) agree on the low-security input variable *l*, globally agree on the low-security output *o*.^1^

**Fig. 1:**
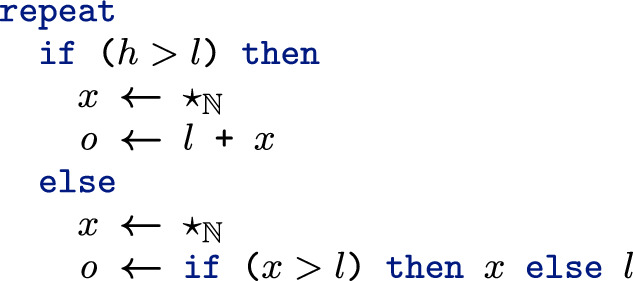
Example program

***Beyond*** *k*-***Safety.***

The vast landscape of hyperproperties does, however, stretch far beyond *k*-safety. The overarching limitation of *k*-safety (or, more generally, of hypersafety [37]) is an implicit *universal* quantification over all executions. By contrast, many properties of interest, including applications in refinement, information-flow control, and robustness, require a combination of universal and existential quantification. As an example, consider the program in Fig. 1, where $\star _{\mathbb {N}}$ denotes a nondeterministic choice of a natural number. We assume that *h*, *l*, and *o* are a high-security input, a low-security input, and a low-security output, respectively. This program violates the simple 2-safety non-interference property given above as the non-determinism influences the value of the output. Nevertheless, the program is *secure* in the sense that an attacker that observes the low-security input and the output cannot deduce information about the high-security input. To see this, assume the attacker observes the low-security inputs and outputs on some trace $\pi$. The key observation is that the (low-security) input–output behavior on $\pi$ is possible for any possible high-security input, i.e., for every possible value of *h*, there *exists* some way to resolve the nondeterminism such that the low-security observations of the attacker agree with the ones made on $\pi$. To capture this formally, we use a relaxed notion of non-interference, in the literature often referred to as generalized non-interference (GNI) [69]. We can express GNI as

$$\begin{aligned} \forall \pi _1\mathpunct {.}\forall \pi _2\mathpunct {.}\exists \pi _3\mathpunct {.}\Box \big ( o_{\pi _1} = o_{\pi _3} \wedge l_{\pi _1} = l_{\pi _3}\big ) \wedge \Box \big (h_{\pi _2} = h_{\pi _3} \big ). \end{aligned}$$

This property requires that for any two traces $\pi _1, \pi _2$, there exists a third trace $\pi _3$ that, globally, agrees with the low-security inputs and outputs on $\pi _1$ but the high-security inputs on $\pi _2$. The program in Fig. 1 satisfies GNI.

***Hyperliveness and*** $\forall ^k\exists ^l$-***Safety.***

Crucially, GNI is no longer a hypersafety property (and, in particular, no *k*-safety property for any *k*) as it requires a combination of universal and *existential* quantification over traces. Instead, GNI is—in the terminology of Clarkson and Schneider [37]—a *hyperliveness* property.^2^ In particular, GNI is what we call a $\forall ^2\exists ^1$-safety property. More generally, for $k, l \in \mathbb {N}$, a $\forall ^k\exists ^l$-*safety property* quantifies universally over *k* traces followed by an existential quantification over *l* traces and states that the resulting $k+l$ traces do not interact badly (i.e., poses a safety requirement on $k+l$ traces). *k*-safety properties are the *special case* where $l = 0$, i.e., *k*-safety properties are $\forall ^k\exists ^0$-safety properties.

***Verification of Temporal Hyperproperties.***

Existing approaches for the verification of temporal hyperproperties impose restrictions on either the class of systems (by, e.g., considering only finite-state transition systems) or the class of specifications (by, e.g., only considering *k*-safety properties): When restricting to *finite-state* systems, the model-checking problem for logics such as HyperLTL is decidable [56] and efficient model-checking tools that can handle quantifier alternations exist [19, 23, 64]. In contrast, when verifying *infinite*-state systems, verification has, so far, been limited to *k*-safety properties [8, 12, 54, 77, 78] or functional (opposed to temporal) $\forall ^k\exists ^l$ properties [14, 49, 79].

### Verification of hyperliveness in infinite-state systems

In this paper, we present a verification technique for $\forall ^k\exists ^l$-safety properties in infinite-state systems. Our novel verification method is based on a combination of **(1)** a game-based reading of existential trace quantification and **(2)** the search of a program reduction. We study this combination in the context of a *predicate abstraction* of the system(s) [58].

***(1) Game-based Reading of Existential Quantification.***

The idea of a game-based reading of existential quantification is to instantiate existentially quantified traces with a *strategy* [17, 22, 38]. When trying to verify a $\forall ^k\exists ^l$ property, we, instead, attempt to find strategies $\sigma _1, \ldots , \sigma _l$ for each of the *l* existentially quantified traces and use the *i*th strategy to resolve the *i*th existentially quantified trace. If we find appropriate strategies (that construct appropriate concrete witnesses for existentially quantified traces), we have verified the $\forall ^k\exists ^l$ property.

***(2) Program Reduction.***

The idea of a program reduction is based on the observation that we can *reorder* independent program instructions [68]. Such a reordering does not change the program’s semantics, but it can make the verification much easier (by, e.g., enabling the use of simpler invariants) [54, 55, 68, 77]. When verifying hyperproperties, we can make use of such reductions to reorder instructions from individual program copies in a self-composition.

***Combining Games and Program Reductions.***

So far, both techniques are limited to their respective domain, i.e., the game-based approach has only been applied to finite-state systems, and reductions have (mostly) been used for the verification of *k*-safety properties. We combine both techniques yielding an effective (and first) verification technique for $\forall ^k\exists ^l$-safety properties in infinite-state systems. Our verification approach works within a fixed *predicate abstraction*, i.e., we track finitely many relational predicates to capture relevant information about the joint state-space of the system copies. Strategies for existentially quantified variables then operate on abstract states (while ensuring that the strategy on abstract states extends to a strategy on concrete states), and reductions increase the precision of the abstraction by admitting alignments (reorderings) that work well within a given set of predicates. Notably, our search for reduction and strategy-based instantiation of existential quantification is *mutually dependent*, i.e., a particular strategy might depend on a particular reduction and vice versa.

### Contributions and structure

***OHyperLTL.***

The starting point of our work is a new temporal logic called *Observation-based HyperLTL* (OHyperLTL for short). Our logic extends the existing hyperlogic HyperLTL [36] with capabilities to reason about asynchronous properties (i.e., properties where the individual traces are traversed at different speeds), and to specify properties using assertions from arbitrary background theories (to reason about the infinite variable domains encountered in, e.g., software) (Sect. 4).

***Reductions as Games.***

To automatically verify $\forall ^k\exists ^l$-safety OHyperLTL properties, we combine program reductions with a strategy-based instantiation of existential quantification, both in the context of a fixed predicate abstraction. To facilitate this combination, we first present a game-based approach that automates the search for a reduction within a fixed set of predicates. Concretely, we construct a game (played on abstract states generated by the given predicates) where a winning strategy for the verifier directly corresponds to a reduction of the system that establishes the safety property (Sect. 5).

***Existential Quantification as Games.***

Our strategic (i.e., game-based) view on reductions allows us to combine them with a game-based instantiation of existential quantification. As alluded to above, we view existentially quantified traces as being constructed by a strategy. As we phrase both the search for a reduction and the search for existentially quantified traces as a game, we can frame the search for both as a combined abstract game. We prove the soundness of our approach, i.e., a winning strategy for the verifier constitutes both a strategy for the existentially quantified traces and accompanying (mutually dependent) reduction (Sect. 6).

***Implementation.***

We have implemented our verification approach in a prototype tool called HyPA (short for **Hy**perproperty Verification with **P**redicate **A**bstraction) and evaluate HyPA on *k*-safety properties (that can already be handled by existing methods) and on $\forall ^k\exists ^l$-safety benchmarks that cannot be handled by any existing tool (Sect. 7).

***Contributions Overview.***

In short, our contributions include the following:

- We propose OHyperLTL, a novel temporal hyperlogic that can specify asynchronous hyperproperties in infinite-state systems;
- We present a game-based interpretation of a reduction within a fixed predicate abstraction;
- We combine a strategy-based instantiation of existentially quantified traces with the search for a reduction; yielding a flexible (and first) method for the verification of temporal $\forall ^k\exists ^l$-safety properties;
- We implement and evaluate a prototype implementation of our method.

***Conference Version.***

This paper is an extended version of a preliminary conference version [18]. Compared to the conference paper, this version adds additional explanations and includes various examples that illustrate key concepts. In addition:

- We give a formal description of all games (Sects. 5.2 and 6.2) and provide algorithms for constructing them (in Sect. 6.7);
- We improve the precision of the abstract games for the verification of $\forall ^k\exists ^l$ properties. Concretely, we propose a mechanisms that allows the verifier to restrict the set of abstract initial states (Sect. 6.3);
- We study the exact relation between our *k*-safety game and our game for $\forall ^k\exists ^l$-safety properties. Using a direct correspondence between the restrictions used in the $\forall ^k\exists ^l$ game and the (standard) abstract transition relation in the *k*-safety game, we show that our game for $\forall ^k\exists ^l$-safety properties *generalizes* the *k*-safety game (Sect. 6.6).

## Overview: reductions and quantifier alternations as a game

Our verification approach hinges on the observation that we can express both the search for a suitable reduction and the search for witness traces for existential quantification as games. In this section, we provide an overview of our game-based interpretations. We begin by outlining our game-based reading of a reduction (illustrating this in the simpler case of *k*-safety) in Sect. 2.1 and then extend this to include a game-based interpretation of existential quantification in Sect. 2.2.

### Reductions as a game

**Fig. 2:**
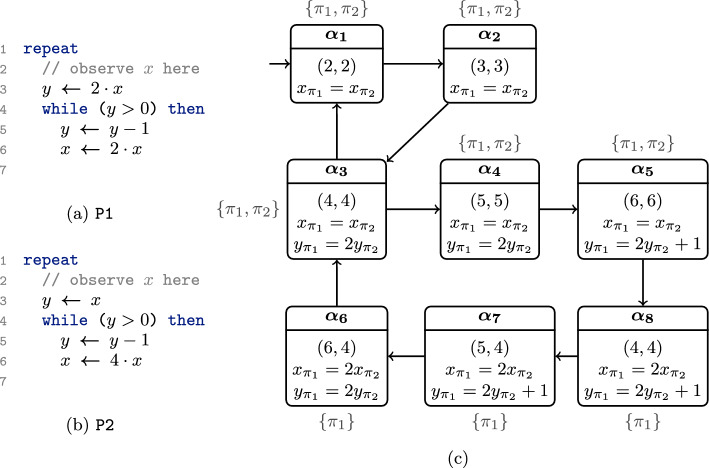
Two programs P1 and P2 are depicted in Fig. 2a and b. In Fig. 2c, (parts of) a winning strategy for the verifier is given. Each state is labeled $\alpha _1, \ldots , \alpha _8$ and contains the value of the program counter of both copies (given as the pair at the top) as well as the predicates that hold in that state. We mark the initial state $\alpha _1$ with an incoming arrow. The outer label at each state gives the scheduling $M \subseteq \{\pi _1, \pi _2\}$ chosen by the strategy in that state

We consider the two programs P1 and P2 in Fig. 2a and b. Assume that, in both programs, we *observe* the program variables whenever the program is at line 2 (think of all variables being printed to the command line at this location). We want to check that if *x* is equal across the two programs at the first observation, then, in all further observations, the value of *x* also coincides. We can formalize this property in our logic OHyperLTL (formally defined in Sect. 4) as follows:

$$\begin{aligned} \forall ^{\texttt {P1}} \, \pi _1 :( pc = 2). \; \forall ^{\texttt {P2}} \, \pi _2 : ( pc = 2). \; (x_{\pi _1} = x_{\pi _2}) \rightarrow \Box (x_{\pi _1} = x_{\pi _2}) \end{aligned}$$

The property states that for all traces $\pi _1$ in P1 and $\pi _2$ in P2 the LTL specification $(x_{\pi _1} = x_{\pi _2}) \rightarrow \Box (x_{\pi _1} = x_{\pi _2})$ holds. Here, $x_{\pi _i}$ refers to the value of *x* on trace $\pi _i$ (for $\pi _i \in \{\pi _1, \pi _2\}$).

In addition, OHyperLTL features so-called *observation points* within its specification. The *observation formula* $pc = 2$ identifies the positions of a trace in which we evaluate (progress) the temporal property. More formally, whenever we quantify over a trace, we first project the trace on those step where $pc = 2$ holds, i.e., steps where the program counter ($pc$) is in line 2. These observation points facilitate the specification of *asynchronous* hyperproperties, i.e., properties where we do not observe the traces in lock-step (we give more details in Sect. 4).

***Self-Composition and Reductions.***

The verification of our property involves reasoning about two copies of our system (in this case, one of P1 and one of P2) on *disjoint* state spaces. Consequently, we can interleave the statements of both programs (between two observation points) without affecting the behavior of the individual copies. We refer to each interleaving of both copies as a *reduction* [68]. The choice of this reduction influences the complexity of the needed invariants [54, 55, 68, 77]. Given a set of relational predicates $\mathcal {P}$, we aim to discover a suitable reduction of the system that proves the property within the abstraction captured by $\mathcal {P}$ [77]. Our first observation is that we can phrase the search for a reduction as a game played between a verifier and a refuter over abstract states as follows. In each step, the verifier decides on a *scheduling* (i.e., a non-empty subset $M \subseteq \{\pi _1, \pi _2\}$) that indicates which of the copies should take a step (i.e., $\pi _i \in M$ iff the copy of program P*i* should make a program step). Afterward, the refuter can choose any abstract successor state that can be reached from the current abstract state under the scheduling picked by the verifier. The process then repeats from this new abstract state. This naturally defines a finite-state two-player safety game that we can solve efficiently.^3^ If the verifier wins, a winning strategy directly corresponds to a reduction and accompanying inductive invariant for the safety property within the predicate abstraction spanned by $\mathcal {P}$.

***Example Strategy.***

For our example, we give (parts of) a possible winning strategy in Fig. 2c. Here, we use predicates $\mathcal {P}= \{x_{\pi _1} = x_{\pi _2}, x_{\pi _1} = 2 x_{\pi _2}, y_{\pi _1} = 2y_{\pi _2}, y_{\pi _1} = 2y_{\pi _2} + 1\}$ and, additionally, track the program location of both programs. We only depict the relevant predicates in each state. In each state, the strategy chooses a scheduling $M \subseteq \{\pi _1, \pi _2\}$ (written next to the state), and all abstract states compatible with that scheduling are listed as successors. Note that whenever both copies are at an observation point (i.e., both programs are in line 2), it holds that $x_{\pi _1} = x_{\pi _2}$ (cf. state $\alpha _1$). The example strategy schedules in lock-step for the most part (by choosing $M = \{\pi _1, \pi _2\}$) but lets P1 take the inner loop *twice*; in state $\alpha _8$ (after both programs have completed the inner loop once), it only schedules $\{\pi _1\}$.^4^ This scheduling allows us to maintain the linear invariants $x_{\pi _1} = x_{\pi _2}$ and $y_{\pi _1} = 2y_{\pi _2}$.

In particular, the resulting reduction is property-based [77], as the scheduling is based on the current (abstract) state. Note that the program cannot be verified with only linear invariants in a sequential or parallel (lock-step) reduction.

### Quantifier alternations as a game

**Fig. 3:**
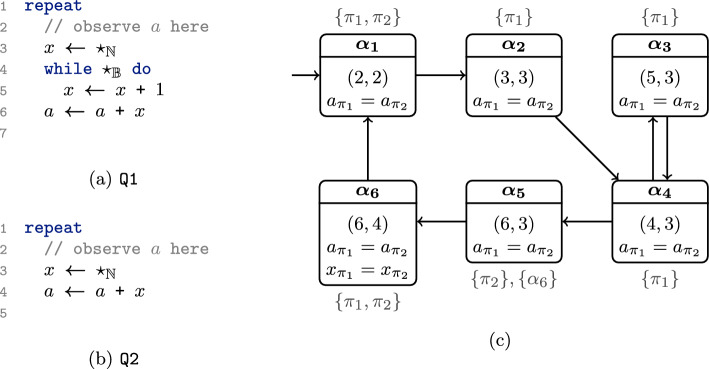
Two programs Q1 and Q2 are given in Fig. 3a and b. In Fig. 3c, (parts of) a winning strategy for the verifier is depicted. The outer label gives the scheduling $M \subseteq \{\pi _1, \pi _2\}$ and, if applicable, the restriction chosen by the witness strategy

We build upon this game-based interpretation of a reduction to move beyond *k*-safety and handle $\forall ^k\exists ^l$-safety properties. As a second example, consider the two programs Q1 and Q2 in Fig. 3a and b, where $\star _\tau$ denotes a nondeterministic choice of type $\tau \in \{\mathbb {N}, \mathbb {B}\}$. We wish to check that Q1 refines Q2, i.e., all output behavior of Q1 is also possible in Q2. We can express this in our logic as follows:

$$\begin{aligned} \forall ^{\texttt {Q1}} \pi _1 : ( pc = 2). \; \exists ^{\texttt {Q2}} \pi _2 : ( pc = 2). \; \Box (a_{\pi _1} = a_{\pi _2}) \end{aligned}$$

The property states that for every trace $\pi _1$ in Q1, there *exists* a trace $\pi _2$ in Q2 that globally agrees on the value of *a*. We, again, make use of observation points and only observe a trace when the program is in line 2 (i.e., $pc = 2$).

The quantifiers range over infinite traces of variable assignments (with infinite domains), making a direct verification of the quantifier alternation challenging. In contrast to alternation-free formulas, we cannot reduce the verification to verification on a self-composition. Instead, we adopt (yet another) game-based interpretation by viewing the existentially quantified traces as being resolved by a *strategy* (called the *witness strategy*) [38]. That is, instead of trying to find a witness trace $\pi _2$ in Q2 when given the *entire* trace $\pi _1$, we interpret the $\forall ^1\exists ^1$ property as a game between verifier and refuter. The refuter moves through the state space of Q1 (thereby producing a trace $\pi _1$), and the verifier reacts to each move by choosing a concrete successor state in the state space of Q2 following the witness strategy (thereby producing a trace $\pi _2$). If the witness strategy can ensure that the resulting traces $\pi _1, \pi _2$ satisfy $\Box (a_{\pi _1} = a_{\pi _2})$, the $\forall ^1\exists ^1$ property holds. Finding a winning strategy for the verifier is difficult when the game progresses *synchronously* (i.e., strictly alternating between progressing $\pi _1$ and $\pi _2$). For example, in Fig. 3 an (informal) solution to construct a witness trace $\pi _2$ (when given the *entire* trace $\pi _1$) would be to guarantee that in Q2:4 (meaning program location 4 of Q2) and Q1:6, the value of *x* in both programs agrees (i.e., $x_{\pi _1} = x_{\pi _2}$ holds). However, to follow this idea, the witness strategy for the verifier, when at Q2:3 (the location where the new value of $x_{\pi _2}$ is chosen), would need to know the *future* value of $x_{\pi _1}$ when Q1 is at location Q1:6.

***Combining Reductions and Witness Strategy.***

Our insight in this paper is that we can turn the strategy-based interpretation of the witness trace $\pi _2$ into a useful verification method by *combining* it with a program reduction. As we express both searches as games, we can phrase the combined search as a combined game. In this game, the verifier chooses a scheduling (as in Sect. 2.1), and, additionally, whenever the existentially quantified copy is scheduled, the verifier also decides on the successor state of that copy. In particular, both the reduction and the witness strategy are controlled by the verifier and can thus *collaborate*.

***Example Strategy.***

We depict (parts of) a winning strategy for the verifier in Fig. 3c. Here, we use predicates $\mathcal {P}= \{a_{\pi _1} = a_{\pi _2}, x_{\pi _1} = x_{\pi _2}\}$ and, additionally, track the program location of both programs. As in Fig. 2c, we only depict the relevant predicates in each state. This strategy formalizes the interplay of reduction and witness strategy. Initially, the verifier only schedules $\{\pi _1\}$ until Q1 has reached Q1:6 and the value of *x* is fixed (in state $\alpha _5$). Once in state $\alpha _5$, the verifier schedules $\{\pi _2\}$. As $\pi _2$ is quantified existentially, the verifier can (via a witness strategy) decide on a successor state for the $\pi _2$-copy. In our case, the verifier chooses a value for $x_{\pi _2}$ such that $x_{\pi _1} = x_{\pi _2}$ holds. As we work in an abstraction of the actual system, we formalize this by restricting the abstract successor states. Concretely, in state $\alpha _5$, the verifier schedules $\{\pi _2\}$ and simultaneously restricts the successors to $\{\alpha _6\}$ (i.e., the abstract state where $x_{\pi _1} = x_{\pi _2}$ holds), even though the abstract state
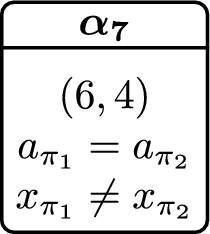


is also a valid successor of $\alpha _5$ under scheduling $\{\pi _2\}$ (note that $\alpha _7$ is not part of Fig. 3c). Intuitively, the verifier can restrict the abstract successor states to be within $\{\alpha _6\}$ as, from all *concrete* states in $\alpha _5$, we can ensure a transition (by controlling the non-deterministic assignment at Q2:3) to a concrete state that abstracts to an abstract state in $\{\alpha _6\}$. We formalize when a restriction is valid in Sect. 6. The resulting strategy is winning and therefore denotes a reduction *and* witness strategy for the existentially quantified copy. Notably, both reduction and witness strategy are mutually dependent, i.e., the restriction chosen by the verifier (corresponding to the choice of a witness function) depends on the current abstract state and the scheduling.

## Preliminaries

We begin by introducing basic preliminaries, including our basic model of computation and background on (finite-state) safety games.

***First-Order Background Theory.***

We assume some fixed underlying first-order theory $\mathfrak {T}$. For the remainder of this paper, we assume (for simplicity) that all variables range over $\mathbb {N}$. Given a first-order formula $\theta$ over free variables *Y* and some assignment $\mu : Y \rightarrow \mathbb {N}$ mapping variables in *Y* to concrete values, we write $\mu \models _\mathfrak {T}\theta$ if $\mu$ satisfies $\theta$ (modulo $\mathfrak {T}$). We write $\texttt {SAT}(\theta )$ if $\theta$ is satisfiable, i.e., there exists some assignment $\mu$ such that $\mu \models _\mathfrak {T}\theta$. Given two assignments $\mu _1: Y_1 \rightarrow \mathbb {N}$ and $\mu _2: Y_2 \rightarrow \mathbb {N}$ over disjoint domains (i.e., $Y_1 \cap Y_2 = \emptyset$), we define $\mu _1 \uplus \mu _2: (Y_1 \uplus Y_2 )\rightarrow \mathbb {N}$ as the union of both assignments.

***Symbolic Transition Systems.***

A *symbolic transition system* (STS) is a tuple $\mathcal {T} = (X, init , step )$ where *X* is a finite set of system variables, $init$ is a first-order formula with free variables from *X* describing all initial states, and $step$ is a formula over $X \uplus X'$ (where $X' :=\{x' \mid x \in X\}$ is the set of primed variables; disjoint from *X*) describing the transitions of the system. Given an assignment $\mu : X \rightarrow \mathbb {N}$ to *X*, we write $\mu ': X' \rightarrow \mathbb {N}$ for the assignment over $X'$ defined by $\mu '(x') :=\mu (x)$. A trace in $\mathcal {T}$ is an infinite sequence of assignment $\mu _0\mu _1\cdots$ such that $\mu _0 \models _\mathfrak {T} init$ and for every $i \in \mathbb {N}$, $\mu _i \uplus \mu _{i+1}' \models _\mathfrak {T} step$. We write $Traces (\mathcal {T})$ for the set of all traces in $\mathcal {T}$. We assume that each state in the system has a successor, i.e., for every assignment $\mu$ to *X*, there exists some $\mu '$ to $X'$ such that $\mu \uplus \mu ' \models _\mathfrak {T} step$.

We can interpret programs as STS by modeling the current program location using a dedicated variable, usually called $pc$.

### Example 1

Consider the program in Fig. 3a. We can model this program as the STS $\mathcal {T}:= (\{a, x, pc \}, init , step )$ modulo linear integer arithmetic (LIA) where $init := (pc = 2)$ and

$$\begin{aligned} step := \,&\big ( pc = 2 \rightarrow ( pc ' = 3 \wedge a' = a \wedge x' = x) \big ) \wedge \\&\big ( pc = 3 \rightarrow ( pc ' = 4 \wedge a' = a) \big ) \wedge \\&\big ( pc = 4 \rightarrow ( pc ' = 5 \wedge a' = a \wedge x' = x) \vee ( pc ' = 6 \wedge a' = a \wedge x' = x) \big ) \wedge \\&\big ( pc = 5 \rightarrow ( pc ' = 4 \wedge a' = a \wedge x' = x + 1) \big ) \wedge \\&\big ( pc = 6 \rightarrow ( pc ' = 2 \wedge a' = a + x \wedge x' = x ) \big ). \end{aligned}$$

***Formula Indexing.***

We fix a finite set of trace variables $\{\pi _1, \ldots , \pi _k\}$ (which we will use in our OHyperLTL specification). For a trace variable $\pi _i \in \{\pi _1, \ldots , \pi _k\}$ we define $X_{\pi _i}$ as an indexed set of variables, i.e., $X_{\pi _i} :=\{x_{\pi _i} \mid x \in X\}$. Similarly, we define $X'_{\pi _i} :=\{x'_{\pi _i} \mid x \in X\}$ as an indexed set of primed variables. We abbreviate $\vec {X}:= X_{\pi _1} \cup \cdots \cup X_{\pi _k}$ and $\vec {X}': = X'_{\pi _1} \cup \cdots \cup X'_{\pi _k}$. For a first-order formula $\theta$ over free variables from $X \cup X'$, we define $\theta _{\langle \pi _i \rangle }$ as the formula over $X_{\pi _i} \cup X_{\pi _i}'$ obtained by replacing every free variable *x* with $x_{\pi _i}$ and $x'$ with $x'_{\pi _i}$. For a first-order formula $\theta$ over $\vec {X}$, we define $\theta ^{\langle '\rangle }$ as the formula over $\vec {X}'$ obtained by replacing every free variable $x_{\pi _i}$ with $x_{\pi _i}'$.

***Safety Games.***

A *safety game* is a tuple $\mathcal {G} = (S_\texttt {SAFE}, S_\texttt {REACH}, s_ {init} , T, S_ {bad} )$ where $S:= S_\texttt {SAFE}\uplus S_\texttt {REACH}$ is a set of game states, $s_ {init} \in S$ is an initial state, $T \subseteq S \times S$ is a transition relation, and $S_ {bad} \subseteq S$ is a set of bad states. We assume that for every $s \in S$, there exists at least one $s'$ with $(s, s') \in T$. States in $S_\texttt {SAFE}$ are controlled by player $\texttt {SAFE}$ and those in $S_\texttt {REACH}$ by player $\texttt {REACH}$. A play is an infinite sequence of states $s_0s_1\cdots$ such that $s_0 = s_ {init}$, and $(s_i, s_{i+1}) \in T$ for every $i \in \mathbb {N}$. A positional strategy $\sigma$ for player $p \in \{\texttt {SAFE}, \texttt {REACH}\}$ is a function $\sigma : S_p \rightarrow S$ such that $(s, \sigma (s)) \in T$ for every $s \in S_p$. A play $s_0s_1\cdots$ is compatible with strategy $\sigma$ for player *p* if $s_{i+1} = \sigma (s_i)$ whenever $s_i \in S_p$. The safety player wins $\mathcal {G}$ if there is a strategy $\sigma$ for $\texttt {SAFE}$ such that all $\sigma$-compatible plays never visit a state in $S_ {bad}$.

## Observation-based HyperLTL

In this section, we present OHyperLTL (short for observation-based HyperLTL). Our logic builds upon HyperLTL [36], which itself extends linear-time temporal logic (LTL) [73] with explicit trace quantification. In OHyperLTL, we include predicates from the background theory (to reason about infinite variable domains) and explicit observations (to express asynchronous properties). Formulas in OHyperLTL are given by the following grammar:

$$\begin{aligned} \varphi&:= \forall \pi _i : \xi \mathpunct {.}\varphi \mid \exists \pi _i : \xi \mathpunct {.}\varphi \mid \psi \\ \psi&:= \theta \mid \lnot \psi \mid \psi _1 \wedge \psi _2 \mid \bigcirc \psi \mid \psi _1~ \mathcal {U}~\psi _2. \end{aligned}$$

Here $\pi _i \in \{\pi _1, \ldots , \pi _k\}$ is a trace variable, $\theta$ is a first-order formula over $\vec {X} = X_{\pi _1} \cup \cdots \cup X_{\pi _k}$, and $\xi$ is a first-order formula over *X* (called the observation formula). We assume that all trace variables from $\{\pi _1, \ldots , \pi _k\}$ occur in the quantifier prefix *exactly* once.

In OHyperLTL, we quantify over traces in the system (subject to some observation constraint $\xi$ which we explain below) followed by an LTL formula. In this LTL formula, the atomic statements are first-order formulas $\theta$ over $\vec {X} = X_{\pi _1} \cup \cdots \cup X_{\pi _k}$, which express (relational) properties on the current step of the traces $\pi _1, \ldots , \pi _k$. We use the standard Boolean connectives $\wedge$, $\rightarrow$, $\leftrightarrow$, and constants $\top , \bot$, as well as the derived LTL operators eventually $\diamondsuit \psi :=\top ~ \mathcal {U}~\psi$, and globally $\Box \psi :=\lnot \diamondsuit \lnot \psi$.

### Remark 1

For the examples in Sect. 2, we annotated quantifiers with an STS to reason about different STSs within the same formula. In the following, we assume, w.l.o.g., that all quantifiers range over the same (fixed) STS to simplify notation.

### Semantics

Recall that a trace *t* is an infinite sequence of assignments to *X*. For $i \in \mathbb {N}$, we write *t*(*i*) to denote the *i*th assignment in *t*. A trace assignment $\Pi$ is a mapping of trace variables $\pi _1, \ldots , \pi _k$ to traces. Given a trace assignment $\Pi$ and $i \in \mathbb {N}$, we define $\Pi _{(i)}$ to be the assignment to $\vec {X}$ given by $\Pi _{(i)}(x_\pi ) :=\Pi (\pi )(i)(x)$, i.e., the value of $x_\pi$ is the value of *x* in the *i*th step on the trace assigned to $\pi$. For the LTL body of an OHyperLTL formula, we then define

$$\begin{aligned} \Pi , i&\models \theta&\text { iff } \quad&\Pi _{(i)} \models _\mathfrak {T}\theta \\ \Pi , i&\models \lnot \psi&\text { iff } \quad&\Pi , i \not \models \psi \\ \Pi , i&\models \psi _1 \wedge \psi _2&\text { iff } \quad&\Pi , i \models \psi _1 \text { and } \Pi , i \models \psi _2\\ \Pi , i&\models \bigcirc \psi&\text { iff } \quad&\Pi , i + 1 \models \psi \\ \Pi , i&\models \psi _1~\mathcal {U}~\psi _2&\text { iff } \quad&\exists j \ge i\mathpunct {.}\Pi , j\models \psi _2 \text { and } \forall i \le k < j\mathpunct {.}\Pi , k \models \psi _1. \end{aligned}$$

***Observations.***

The distinctive feature of OHyperLTL over HyperLTL are the explicit observations. Given an observation formula $\xi$ (a first-order formula over *X*) and trace *t*, we say that $\xi$ is a *valid observation on* *t*, written $valid (t,\xi )$, if there exist infinitely many $i \in \mathbb {N}$ such that $t(i) \models _\mathfrak {T}\xi$. If $valid (t,\xi )$ holds, we write $\llparenthesis t\rrparenthesis _{\xi }$ for the trace obtained by projecting on those positions *i* where $t(i) \models _\mathfrak {T}\xi$, i.e., $\llparenthesis t\rrparenthesis _{\xi }(i) :=t(j)$ where *j* is the *i*th smallest index that satisfies $\xi$. For a trace assignment $\Pi$, a trace variable $\pi$, and a trace *t*, we define $\Pi [\pi \mapsto t]$ as the updated trace assignment that maps $\pi$ to *t*. Given a set of traces $\mathbb {T}$, we resolve trace quantification as follows:

$$\begin{aligned} \Pi&\models _{\mathbb {T}} \psi&\text { iff } \quad&\Pi , 0 \models \psi \\ \Pi&\models _{\mathbb {T}} \forall \pi : \xi \mathpunct {.}\varphi&\text { iff } \quad&\forall t \in \big \{t \in \mathbb {T}\mid valid (t,\xi )\big \}\mathpunct {.}\Pi \big [\pi \mapsto \llparenthesis t\rrparenthesis _{\xi }\big ] \models _{\mathbb {T}} \varphi \\ \Pi&\models _{\mathbb {T}} \exists \pi : \xi \mathpunct {.}\varphi&\text { iff } \quad&\exists t \in \big \{t \in \mathbb {T}\mid valid (t,\xi )\big \}\mathpunct {.}\Pi \big [\pi \mapsto \llparenthesis t\rrparenthesis _{\xi }\big ] \models _{\mathbb {T}} \varphi . \end{aligned}$$

The semantics mostly agrees with that of HyperLTL [36] but projects each trace to the positions where the observation formula holds. Given an STS $\mathcal {T}$ and OHyperLTL formula $\varphi$, we write $\mathcal {T} \models \varphi$ if $\emptyset \models _{ Traces (\mathcal {T})} \varphi$ where $\emptyset$ is the empty trace assignment.

### Expressiveness

***OHyperLTL and Asynchronous Hyperproperties.***

The explicit observations in OHyperLTL facilitate the specification of asynchronous hyperproperties, i.e., properties where traces are traversed at different speeds. For example, in Sect. 2.1, the explicit observations allow us to compare the variables of both programs at line 2 even though the actual step at which line 2 is reached (in a synchronous semantics) differs between both programs (as P1 takes the inner loop twice as often as P2). As the observations are part of the specification, we can model a broad spectrum of properties ranging, e.g., from time-insensitive properties (by placing observations only at locations that are observable by an attacker, such as, e.g., output statements) to time-sensitive specifications [57] (by placing observations at every program location).

***Functional Specification in OHyperLTL.***

OHyperLTL is intended to express *temporal* properties (i.e., properties that reason about infinite executions) but can also express *functional* *k*-safety requirements. A functional *k*-safety specification is given by a precondition $\theta _{ pre }$ and postcondition $\theta _{ post }$ as first-order formulas over $\vec {X} = X_{\pi _1} \cup \cdots \cup X_{\pi _k}$. The specification $(\theta _{ pre },\theta _{ post })$ then states that any *k* program executions $\pi _1, \ldots , \pi _k$, when starting in states related by $\theta _{ pre }$ only terminate in states related by $\theta _{ post }$. See, e.g., [77–79] for details. We can easily express this in OHyperLTL: We translate the program into an STS and design an observation formula $\xi$ that only holds in the initial state of the system and in those states where the program has terminated. The functional specification is then expressible in OHyperLTL as

$$\begin{aligned} \forall \pi _1 : \xi \ldots \forall \pi _k : \xi \mathpunct {.}\theta _{ pre } \rightarrow \bigcirc \theta _{ post }. \end{aligned}$$

***OHyperLTL and HyperLTL.***

If we set the observation formula to always hold (i.e, $\xi :=\top$), we observe every step on the trace and can express synchronous properties (note that $\llparenthesis t\rrparenthesis _{\top } = t$ for every trace *t*). OHyperLTL thus subsumes HyperLTL [36]. Note that even when setting $\xi = \top$, OHyperLTL can still reason about infinite variable domains in the background theory (which HyperLTL cannot).

### Finite-state model checking

Many mechanisms used to express asynchronous hyperproperties render finite-state model checking undecidable [11, 30, 59]. In contrast, the simple observation-based mechanism used in OHyperLTL admits decidable finite-state model checking.

#### Theorem 1

Assume an STS $\mathcal {T}$ with finite variable domains and decidable background theory, and an OHyperLTL formula $\varphi$. It is decidable if $\mathcal {T} \models \varphi$.

#### Proof

Under the assumptions, we can view $\mathcal {T}$ as an explicit (instead of symbolic) *finite-state* transition system. Given an observation formula $\xi$, we can effectively compute an explicit finite-state system $\mathcal {T}'$ such that $Traces (\mathcal {T}') = \{ \llparenthesis t\rrparenthesis _{\xi } \mid t \in Traces (\mathcal {T}) \wedge valid (t,\xi )\}$. This reduces OHyperLTL model checking on $\mathcal {T}$ to HyperLTL model checking on the finite explicit-state $\mathcal {T}'$, which is decidable [36]. $\square$

Note that for infinite-state (symbolic) systems, we cannot effectively compute $\mathcal {T}'$ as in the proof of Theorem 1. In fact, there may not even exist a system $\mathcal {T}'$ with the desired property that is expressible in the same background theory:

#### Example 2

Consider the program in Fig. 2a with the observation formula $\xi := ( pc = 2)$. We can easily express this program as an STS $\mathcal {T}$ over linear integer arithmetic (LIA) (similar to Example 1). However, if we want to eliminate asynchronous reasoning (following the proof of Theorem 1), we need to summarize all computations performed between two visits to program location 2 into a single step. For example, if we are only interested in the value of *x* (cf. Sect. 2.1), we obtain the STS $\mathcal {T}' = (\{x\}, init , step )$ where $init := \top$ and $step :=( x' = x \cdot 4^x)$, which is not expressible in LIA.

The finite-state result in Theorem 1 is, therefore, of little relevance for the present paper. Nevertheless, it indicates that our logic is well suited for the automated verification of infinite-state systems as the (inevitable) undecidability stems from the infinite domains in programs and not already from the logic itself.

### Safety fragment of OHyperLTL

In this paper, we only consider OHyperLTL specifications which are temporally safe [15], i.e., formulas where the LTL body denotes a *safety property*. Note that, as we support quantifier alternation, we can still express hyperliveness properties [37, 38]. For example, GNI [69], non-inference [70], and refinement are hyperliveness properties but can be expressed as temporally safe OHyperLTL formulas. We model the LTL body of a formula (which uses first-order atoms over $\vec {X}$) by a *symbolic safety automaton* [43] over $\vec {X}$. Such an automaton is a tuple $\mathcal {A}= (Q, q_0, \delta , B)$ where *Q* is a finite set of states, $q_0 \in Q$ is the initial state, $B \subseteq Q$ is a set of bad states, and $\delta$ is a *finite* set of automaton edges of the form $(q, \theta , q')$ where $q, q' \in Q$ are states and $\theta$ is a formula over $\vec {X}$. Given a trace *t* over assignments to $\vec {X}$, a run of $\mathcal {A}$ on *t* is an infinite sequence of states $q_0q_1\cdots$ (starting in $q_0$) such that for every $i \in \mathbb {N}$, there exists an edge $(q_i, \theta , q_{i+1}) \in \delta$ with $t(i) \models _\mathfrak {T}\theta$. A word is accepted by $\mathcal {A}$ if it has *no* run that visits a state in *B*. The automaton is *deterministic* if for every state $q \in Q$ and every assignment $\mu$ to $\vec {X}$, there exists *exactly one* edge $(q, \theta , q') \in \delta$ with $\mu \models _\mathfrak {T}\theta$.

**Fig. 4:**
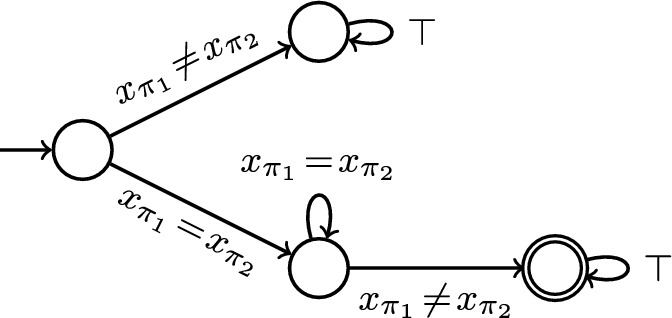
Symbolic safety automaton. We mark the initial state with an incoming arrow and bad states with a double circle

#### Example 3

Consider the example property from Sect. 2.1 with temporal body $(x_{\pi _1} = x_{\pi _2}) \rightarrow \Box (x_{\pi _1} = x_{\pi _2})$. We can translate this LTL formula to the deterministic symbolic safety automaton depicted in Fig. 4.

## Verification of *k*-safety

After having defined our temporal logic, we turn our attention to the automatic verification of OHyperLTL formulas on STSs. In this section, we begin by formalizing our game-based interpretation of a reduction. To illustrate this, we consider $\forall ^k$ OHyperLTL formulas, which, as the body of the formula is a safety property, always denote *k*-safety properties. Let $\mathcal {T} = (X, init , step )$ be an STS, and let

$$\begin{aligned} \varphi = \forall \pi _1 : \xi _1 \ldots \forall \pi _k : \xi _k\mathpunct {.}\psi \end{aligned}$$

be the OHyperLTL we wish to verify. We first translate the LTL formula $\psi$ to a deterministic symbolic safety automaton $\mathcal {A}_\psi = (Q_\psi , q_{\psi , 0}, \delta _\psi , B_\psi )$ over $\vec {X} = X_{\pi _1} \cup \cdots \cup X_{\pi _k}$.

### Predicate abstraction

Our search for a reduction is based in the scope of a fixed predicate abstraction [58, 66]. That is, we abstract our system by keeping track of the truth value of a few selected predicates that (ideally) identify properties that are relevant to prove the property in question.

***Relational Predicates.***

In our abstraction, we capture properties over the combined state space of the *k* system copies that we use to resolve traces $\pi _1, \ldots , \pi _k$. We capture these properties via *relational predicates*, which are first-order formulas over $\vec {X} = X_{\pi _1} \cup \cdots \cup X_{\pi _k}$. Let $\mathcal {P} = \{p_1, \ldots , p_n\}$ be a finite set of relational predicates. We say a formula over $\vec {X}$ is *expressible in* $\mathcal {P}$ if it is equivalent to a boolean combination of the predicates in $\mathcal {P}$. For example, the formula $x_{\pi _1} = x_{\pi _2}$ is expressible in $\{x_{\pi _1} \ge x_{\pi _2}, x_{\pi _1} \le x_{\pi _2}\}$ and expressible in $\{x_{\pi _1} = x_{\pi _2}\}$, but not expressible in $\{x_{\pi _1} = 1, x_{\pi _2} = 1\}$. Any formula $\theta$ that is expressible in $\mathcal {P}$ can be evaluated precisely w.r.t. $\mathcal {P}$, i.e., by only knowing the evaluation of the predicates in $\mathcal {P}$ we can conclude whether or not $\theta$ holds. We assume that all edge formulas in the automaton $\mathcal {A}_\psi$ and all observation formulas $(\xi _{i})_{\langle \pi _i \rangle }$ for $\pi _i \in \{\pi _1, \ldots , \pi _k\}$ are expressible in $\mathcal {P}$. Note that we can always add missing predicates to $\mathcal {P}$ to ensure that all those formulas are expressible.

***Abstract States.***

Given the set of predicates $\mathcal {P}= \{p_1, \ldots , p_n\}$, the state-space of the abstraction w.r.t. $\mathcal {P}$ is given by $\mathbb {B}^n$. For $\hat{s} \in \mathbb {B}^n$ and $1 \le i \le n$ we write $\hat{s}[i] \in \mathbb {B}$ to refer to the *i*th position in $\hat{s}$. Intuitively, $\hat{s}[i]$ tracks whether or not predicate $p_i$ holds. To simplify notation, we define $ite (b, \theta , \theta ')$ (short for if-then-else) as a shorthand for $\theta$ if *b* holds and $\theta '$ otherwise. For each abstract state $\hat{s} \in \mathbb {B}^n$, we then define

$$\begin{aligned} \llbracket \hat{s} \rrbracket :=\bigwedge _{i = 1}^n ite \big (\hat{s}[i] , p_i, \lnot p_i\big ). \end{aligned}$$

That is, $\llbracket \hat{s} \rrbracket$ is a formula over $\vec {X}$ that captures all concrete states that are abstracted to $\hat{s}$. We say that $\hat{s}$ is *non-empty* if $\llbracket \hat{s} \rrbracket$ is satisfiable, i.e., there exists at least one concrete state within $\hat{s}$.

***Transition Relation.***

To incorporate reductions in our abstraction, we parametrize the abstract transition relation by a *scheduling* $M \subseteq \{\pi _1, \ldots , \pi _k\}$. We lift the $step$ formula from $\mathcal {T}$ by defining

$$\begin{aligned} step _M :=\bigwedge _{i = 1}^k ite \Big (\pi _i \in M, step _{\langle \pi _i \rangle }, \bigwedge _{x \in X} x_{\pi _i}' = x_{\pi _i}\Big ). \end{aligned}$$

That is all copies in *M* take a step while all other copies remain unchanged. Recall that $step _{\langle \pi _i \rangle }$ is a formula over $X_{\pi _i} \cup X'_{\pi _i}$. Given two abstract states $\hat{s}_1, \hat{s}_2$, we say that $\hat{s}_2$ is an *M*-*successor* of $\hat{s}_1$, written $\hat{s}_1 \xrightarrow {M} \hat{s}_2$, if $\llbracket \hat{s}_1 \rrbracket \wedge \llbracket \hat{s}_2 \rrbracket ^{\langle '\rangle } \wedge step _M$ is satisfiable, i.e., we can transition from some concrete state in $\hat{s}_1$ to some concrete state in $\hat{s}_2$ by only progressing the copies that are scheduled in *M*.

***Observations in Abstract States.***

During our game construction, we need to check which copy has reached an observation point. We define $obs (\hat{s}) \in \mathbb {B}^k$ as the boolean vector that indicates which copy (of $\pi _1, \ldots , \pi _k$) is currently at an observation point, i.e., which of the observation formulas $\xi _1, \ldots , \xi _k$ hold in $\hat{s}$. Formally, we set $obs (\hat{s})[i] = \top$ iff $\llbracket \hat{s} \rrbracket \wedge (\xi _{i})_{\langle \pi _i \rangle }$ is satisfiable. Recall that we assume that $(\xi _{i})_{\langle \pi _i \rangle }$ is expressible in $\mathcal {P}$. Consequently, either all or none of the concrete states in $\llbracket \hat{s} \rrbracket$ satisfy $(\xi _{i})_{\langle \pi _i \rangle }$.

***Automaton Steps in Abstract States.***

Similarly, we assumed that all automaton edges in $\mathcal {A}_\psi$ are expressible in $\mathcal {P}$. For automaton state $q \in Q_\psi$ and non-empty abstract state $\hat{s}$, we can therefore define $\delta _\psi (q, \hat{s}) \in Q_\psi$ as the *unique* state $q'$ such that there exists an edge $(q, \theta , q') \in \delta _\psi$ such that $\llbracket \hat{s} \rrbracket \wedge \theta$ is satisfiable. Intuitively, this is the unique successor state in $\mathcal {A}_\psi$ that *all* concrete states in $\llbracket \hat{s} \rrbracket$ reach from *q*. Here, the uniqueness follows from the assumption that edge formulas in $\mathcal {A}_\psi$ are expressible in $\mathcal {P}$ (so either all or none of the concrete states in $\llbracket \hat{s} \rrbracket$ satisfy an edge formula) and $\mathcal {A}_\psi$ is deterministic.

### Game construction

Building on the parametrized abstract transition relation, we can construct a (finite-state) safety game where winning strategies for the verifier correspond to reductions that establish the safety property within the precision captured by $\mathcal {P}$.

#### Game states

The states in our game have three forms:

- $(\hat{s}, q, b)$ where $\hat{s} \in \mathbb {B}^n$ is a *non-empty* abstract state (i.e., $\llbracket \hat{s} \rrbracket$ is satisfiable), $q \in Q_\psi$ is a state of the safety automaton for $\psi$, and $b \in \mathbb {B}^k$ is a boolean vector indicating which copy has moved since the last automaton step;
- $(\hat{s}, q, b, M)$ where $\hat{s}$, *q*, and *b* are as before and $\emptyset \ne M \subseteq \{\pi _1, \ldots , \pi _k\}$ is a scheduling;
- $s_ {init}$ which serves as the initial state of the game.

States $(\hat{s}, q, b)$ capture an abstract state $\hat{s} \in \mathbb {B}^n$ of the system and the current state $q \in Q_\psi$ of the safety automaton reached on the previous execution. The boolean vector $b \in \mathbb {B}^k$ records which of the copies has moved since the last observation. We use this information to enforce that no copy is moved even though it has already reached an observation point, i.e., we ensure that all copies *synchronize* on the observation points. States of the form $(\hat{s}, q, b)$ are controlled by player $\texttt {SAFE}$, who takes the role of the verifier. States $(\hat{s}, q, b, M)$, additionally, track a scheduling *M*. These states are controlled by player $\texttt {REACH}$, who takes the role of the refuter. State $s_ {init}$ serves as the initial state of the game and is controlled by $\texttt {REACH}$.

Formally we define the set of states as

1 $$\begin{aligned} \begin{aligned} S_\texttt {SAFE}:= \Big \{(\hat{s}, q, b) \mid \hat{s} \in \mathbb {B}^n \wedge q \in Q_\psi \wedge b \in \mathbb {B}^k \wedge \texttt {SAT}(\llbracket \hat{s} \rrbracket ) \Big \} \end{aligned} \end{aligned}$$

and

2 $$\begin{aligned} \begin{aligned} S_\texttt {REACH}:= \,&\Big \{(\hat{s}, q, b, M) \mid \hat{s} \in \mathbb {B}^n \wedge q \in Q_\psi \wedge b \in \mathbb {B}^k \, \wedge \\&\quad \quad \quad \emptyset \ne M \subseteq \{\pi _1, \ldots , \pi _k \} \wedge \texttt {SAT}(\llbracket \hat{s} \rrbracket ) \Big \} \, \cup \big \{s_{ init } \big \}. \end{aligned} \end{aligned}$$

We define $S = S_\texttt {SAFE}\cup S_\texttt {REACH}$ as the set of all states.

#### Game transitions

**Fig. 5:**
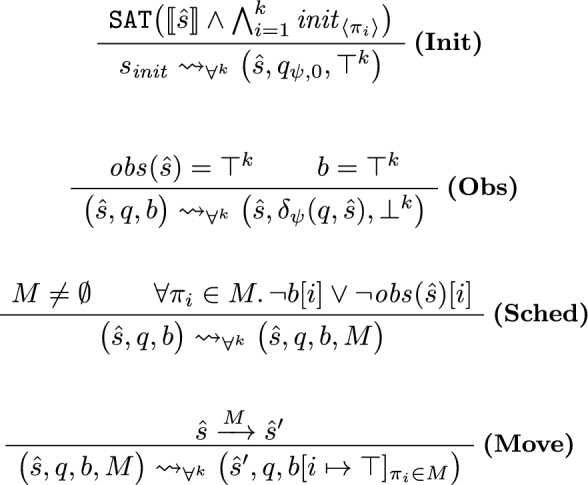
Transition rules for the verification of $\forall ^k$ properties

The transition relation of our game is given via the rules in Fig. 5. We distinguish between four different kinds of transitions, each represented by one of the rules.

***Picking an Initial State (Init).***

Rule **(Init)** is responsible for picking an initial abstract state for the game. As $s_ {init}$ is controlled by $\texttt {REACH}$ (the refuter), $\texttt {REACH}$ can pick any abstract state $\hat{s}$ such that $\llbracket \hat{s} \rrbracket \wedge \bigwedge _{i=1}^k init _{\langle \pi _i \rangle }$ is satisfiable, i.e., any abstract state that contains at least one concrete initial state. The automaton state is initially set to $q_{\psi , 0}$ (the initial state of $\mathcal {A}_\psi$). The boolean vector that tracks which copy has moved since the last update is set to $\top ^k$.

***Updating the Automaton State (Obs).***

In rule **(Obs)**, all copies reached an observation point ($obs (\hat{s}) = \top ^k$) *and* have moved since the last update ($b = \top ^k$). Following the semantics of OHyperLTL, all copies have thus reached a state where the LTL body $\psi$ is progressed, so we update the state of the safety automaton $\mathcal {A}_\psi$. Recall that $\delta _\psi (q,\hat{s})$ is the unique automaton state reached from all concrete states in $\hat{s}$. Moreover, we reset *b* to $\bot ^k$ to record that none of the copies has taken a step since the last automaton update.

***Picking a Scheduling (Sched).***

In rule **(Sched)**, we select any scheduling $M \ne \emptyset$ that schedules only copies that have not reached an observation point or have not moved since the last automaton step. In particular, we cannot schedule any copy that has moved and already reached an observation point. This ensures that all copies always align (i.e., synchronize) at the observation points. States of the form $(\hat{s}, q, b)$ are controlled by the verifier ($\texttt {SAFE}$), who can thus use **(Sched)** to decide which (valid) scheduling should be taken in the current abstract state. Note that either **(Sched)** or **(Obs)** is applicable in any state $(\hat{s}, q, b)$.

***Moving the Copies (Move).***

Lastly, rule **(Move)** is applicable in states of the form $(\hat{s}, q, b, M)$. We update the abstract state to some *M*-successor of $\hat{s}$, i.e., any abstract state that can be reached from some concrete state in $\hat{s}$ by only moving copies scheduled in *M*. Moreover, we update *b* by mapping all copies that took part in the step (i.e., are contained in *M*) to $\top$. States of the form $(\hat{s}, q, b, M)$ are controlled by the refuter ($\texttt {REACH}$), who can thus use **(Move)** to select any (abstract) transition in the system scheduled by *M*.

***All Transitions.***

We define the set of all transition pairs as all transitions that can be derived with $\rightsquigarrow _{\forall ^k}$ using the rules in Fig. 5, i.e.,

3 $$\begin{aligned} T := \big \{(\textbf{x}, \textbf{y}) \in S \times S \mid \textbf{x} \rightsquigarrow _{\forall ^k} \textbf{y}\big \}. \end{aligned}$$

#### Losing game states

We mark a state $(\hat{s}, q, b)$ or $(\hat{s}, q, b, M)$ as losing iff $q \in B_\psi$, i.e., the current state tracking $\mathcal {A}_\psi$ is losing. Formally,

4 $$\begin{aligned} S_ {bad} := \big \{(\hat{s}, q, b) \in S \mid q \in B_\psi \big \} \cup \big \{(\hat{s}, q, b, M) \in S \mid q \in B_\psi \big \}. \end{aligned}$$

#### $\mathcal {G}^\forall _{(\mathcal {T}, \varphi , \mathcal {P})}$

Finally, we define the finite-state safety game $\mathcal {G}^\forall _{(\mathcal {T}, \varphi , \mathcal {P})}$:

##### Definition 1

Define the safety game $\mathcal {G}^\forall _{(\mathcal {T}, \varphi , \mathcal {P})}$ as

$$\begin{aligned} \mathcal {G}^\forall _{(\mathcal {T}, \varphi , \mathcal {P})} := \big (S_\texttt {SAFE}, S_\texttt {REACH}, s_ {init} , T, S_ {bad} \big ) \end{aligned}$$

where $S_\texttt {SAFE}$, $S_\texttt {REACH}$, *T*, and $S_ {bad}$ are defined in (1), (2), (3), and (4), respectively.

### Soundness

If $\texttt {SAFE}$ wins $\mathcal {G}^\forall _{(\mathcal {T}, \varphi , \mathcal {P})}$, a winning strategy picks, in each abstract state, a valid scheduling such that no concrete transition under that scheduling can reach a bad state in $\mathcal {A}_\psi$. We can show that this implies that $\mathcal {T}$ satisfies $\varphi$.

#### Theorem 2

If player $\texttt {SAFE}$ wins $\mathcal {G}^\forall _{(\mathcal {T}, \varphi , \mathcal {P})}$, then $\mathcal {T}\models \varphi$.

#### Proof

Assume $\sigma$ is a winning strategy for $\texttt {SAFE}$ in $\mathcal {G}^\forall _{(\mathcal {T}, \varphi , \mathcal {P})}$. Let $t_1, \ldots , t_k \in Traces (\mathcal {T})$ be arbitrary. We, iteratively, construct stuttered versions $t'_1, \ldots , t'_k$ of $t_1, \ldots , t_k$ by querying $\sigma$ on abstracted prefixes of $t_1, \ldots , t_k$: Whenever $\sigma$ schedules copy *i*, we take a proper step on $t_i$; otherwise, we stutter. By construction of $\mathcal {G}^\forall _{(\mathcal {T}, \varphi , \mathcal {P})}$, the stuttered traces $t'_1, \ldots , t'_k$ align at observation points. In particular, we have $[\pi _1 \mapsto \llparenthesis t_1\rrparenthesis _{\xi _1}, \ldots , \pi _k \mapsto \llparenthesis t_k\rrparenthesis _{\xi _k}] \models \psi$ iff $[\pi _1 \mapsto \llparenthesis t'_1\rrparenthesis _{\xi _1}, \ldots , \pi _k \mapsto \llparenthesis t'_k\rrparenthesis _{\xi _k}] \models \psi$. Moreover, the sequence of abstract states in $\mathcal {G}^\forall _{(\mathcal {T}, \varphi , \mathcal {P})}$ forms an abstraction of $t'_1, \ldots , t'_k$ and shows that $\mathcal {A}_\psi$ cannot reach a bad state when reading $\llparenthesis t'_1\rrparenthesis _{\xi _1}, \ldots , \llparenthesis t'_k\rrparenthesis _{\xi _k}$ (as $\sigma$ is winning). This already shows that $[\pi _1 \mapsto \llparenthesis t'_1\rrparenthesis _{\xi _1}, \ldots , \pi _k \mapsto \llparenthesis t'_k\rrparenthesis _{\xi _k}] \models \psi$ and thus $[\pi _1 \mapsto \llparenthesis t_1\rrparenthesis _{\xi _1}, \ldots , \pi _k \mapsto \llparenthesis t_k\rrparenthesis _{\xi _k}] \models \psi$. As this holds for all traces $t_1, \ldots , t_k \in Traces (\mathcal {T})$, we get $\mathcal {T}\models \varphi$ as required. $\square$

### Constructing and solving $\mathcal {G}^\forall _{(\mathcal {T}, \varphi , \mathcal {P})}$

If the background theory is decidable, we can construct $\mathcal {G}^\forall _{(\mathcal {T}, \varphi , \mathcal {P})}$ using at most $\mathcal {O}(2^{|\mathcal {P}|} \cdot 2^{|\mathcal {P}|} \cdot 2^k)$ queries to an SMT solver; for all valid schedulings $M \subseteq \{\pi _1, \ldots , \pi _k\}$ we determine all *M*-successors for all abstract states (of which there are $2^{|\mathcal {P}|}$ many). Once $\mathcal {G}^\forall _{(\mathcal {T}, \varphi , \mathcal {P})}$ is computed, we can solve this (finite-state) safety game in linear time.

#### Remark 2

Our game-based approach also applies to non-relational verification. Let $\varphi = \forall \pi _1: \top \mathpunct {.}\psi$ be some *synchronous* $\forall ^1$ OHyperLTL property, i.e., we observe the trace at all times by setting the observation formula to $\top$. In the resulting game $\mathcal {G}^\forall _{(\mathcal {T}, \varphi , \mathcal {P})}$, each game state $(\hat{s}, q, b)$ admits a unique valid scheduling (namely $\{\pi _1\}$), so there exists a unique strategy for $\texttt {SAFE}$. In particular, $\texttt {SAFE}$ wins $\mathcal {G}^\forall _{(\mathcal {T}, \varphi , \mathcal {P})}$ iff we can establish that $\mathcal {T}$ satisfies the safety property $\psi$ using standard (non-relational) abstraction w.r.t. the same set of predicates $\mathcal {P}$ [58]. In this case, constructing $\mathcal {G}^\forall _{(\mathcal {T}, \varphi , \mathcal {P})}$ requires $\mathcal {O}(2^{|\mathcal {P}|} \cdot 2^{|\mathcal {P}|})$ SMT queries, the same as (standard) predicate abstraction [58].

***Lazy Solving.***

When constructing the game $\mathcal {G}^\forall _{(\mathcal {T}, \varphi , \mathcal {P})}$ in its entirety, we explore all possible scheduling in all possible abstract states. However, if $\texttt {SAFE}$ wins $\mathcal {G}^\forall _{(\mathcal {T}, \varphi , \mathcal {P})}$, any winning strategy will only pick one concrete scheduling in each state, so some *subgame* will already suffice to establish that $\texttt {SAFE}$ wins $\mathcal {G}^\forall _{(\mathcal {T}, \varphi , \mathcal {P})}$. Based on this idea, we can use a *lazy* algorithm to solve $\mathcal {G}^\forall _{(\mathcal {T}, \varphi , \mathcal {P})}$ by only exploring one valid scheduling in each abstract state (guided by some heuristic). If we hash all intermediate SMT queries, this requires at most $\mathcal {O}(2^{|\mathcal {P}|} \cdot 2^{|\mathcal {P}|} \cdot 2^k)$ SMT queries, i.e., it can never perform worse than constructing the entire game directly.

## Verification of $\forall ^k\exists ^l$-safety

Building on the game-based interpretation of a reduction, we extend our verification beyond $\forall ^k$ properties to support $\forall ^k\exists ^l$-safety properties. We accomplish this by combining the game-based reading of a reduction (as discussed in the previous section) with a game-based reading of existential quantification. For the remainder of this section, we fix an STS $\mathcal {T}= (X, init , step )$ and let

$$\begin{aligned} \varphi = \forall \pi _1 : {\xi _1} \ldots \forall \pi _k : {\xi _k}\mathpunct {.}\exists \pi _{k+1} : {\xi _{k+1}} \ldots \exists \pi _{k+l} : {\xi _{k+l}}\mathpunct {.}\psi \end{aligned}$$

be the OHyperLTL formula we wish to verify. Note that, as in Sect. 5, we assume that $\psi$ denotes a safety property. That is, our verification method, currently, only applies to $\forall ^k\exists ^l$-*safety* properties (such as GNI [69], non-inference [70], and refinement); not arbitrary $\forall ^k\exists ^l$ properties. We further assume that for every existential quantification $\exists \pi _i: \xi _i$ occurring in $\varphi$, $valid (t,\xi _i)$ holds for every $t \in Traces (\mathcal {T})$, i.e., all traces visit observation points infinitely often (we discuss this later in Remark 3).

As in Sect. 5, we work with a fixed set $\mathcal {P}= \{p_1, \ldots , p_n\}$ of relational predicates over $\vec {X}:= X_{\pi _1} \cup \cdots \cup X_{\pi _{k+l}}$ which capture properties over the combined state-space of $k+l$ system copies. We, again, assume that $\mathcal {A}_\psi = (Q_\psi , q_{\psi , 0}, \delta _\psi , B_\psi )$ is a deterministic symbolic safety automaton over $\vec {X}$ for $\psi$.

### Existential trace quantification as a game

The idea of a game-based interpretation of existential trace quantification is to consider the verification as a game between a verifier and a refuter [17, 38]. The refuter controls the *k* universally quantified traces by moving through *k* copies of the system (thereby producing traces $\pi _1, \ldots , \pi _k$), and the verifier reacts by, incrementally, moving through *l* copies of the system (thereby producing traces $\pi _{k+1}, \ldots , \pi _{k+l}$). If the verifier has a strategy that ensures that the resulting traces satisfy $\psi$, we can conclude that $\mathcal {T}\models \varphi$ [17]. We call such a strategy a *witness strategy*.

***Scheduling and Witness Strategies on Concrete States.***

We combine this game-based reading of existential quantification with our game-based interpretation of a reduction by additionally letting the verifier control the scheduling of the system. Let us consider this game at the level of *concrete* states. We start in some concrete state (i.e., some assignment to $\vec {X} = X_{\pi _1} \cup \cdots \cup X_{\pi _{k+l}}$) and proceed in three stages: **(1)** The verifier selects a valid scheduling $M \subseteq \{\pi _1, \ldots , \pi _{k+l}\}$; **(2)** The refuter selects successor states for all universally quantified copies by fixing an assignment to $X_{\pi _1}', \ldots , X_{\pi _k}'$ (under the assumption that only copies scheduled in *M* perform a proper step); **(3)** The verifier reacts by choosing successor states for the existentially quantified copies by fixing an assignment to $X_{\pi _{k+1}}', \ldots , X_{\pi _{k+l}}'$ (again, only moving copies scheduled by *M*). Afterward, the process repeats from the updated concrete state (the assignment over $\vec {X}' = X'_{\pi _1} \cup \cdots \cup X'_{\pi _{k+l}}$).

***Scheduling and Witness Strategies on Abstract States.***

As we work within a predicate abstraction of $\mathcal {T}$, the verifier can, however, not choose concrete successor states directly but only work in the precision captured by $\mathcal {P}$. Following the general scheme of abstract games, we, therefore, underapproximate the moves available to the verifier [46]. Formally, we abstract the three-stage game outlined before (which was played at the level of concrete states) to a simpler abstract game (consisting of only two stages). In the first stage, the verifier selects both a scheduling *M* and a *restriction* *A*. Here, a restriction is a subset $A \subseteq \mathbb {B}^n$ of abstract states that limits the available abstract successor states. In the second stage, the refuter cannot choose any abstract successor state (any *M*-successor in the terminology from Sect. 5), but only abstract states contained in the restriction *A*. To guarantee the soundness of this approach, we ensure that the verifier can only pick restrictions that are *valid*, i.e., restrictions that underapproximate the possibilities of the verifier on the level of concrete states.

### Game construction

To incorporate the idea of restrictions in our game, we build upon the game used in Sect. 5 for *k*-safety verification.

#### Game states

States in our new game now have four different forms:

- $$(\hat{s}, q, b)$$ where $$\hat{s} \in \mathbb {B}^n$$, $$q \in Q_\psi$$, and $$b \in \mathbb {B}^{k+l}$$;
- $$(\hat{s}, q, b, M, A)$$ where $$\hat{s} \in \mathbb {B}^n$$, $$q \in Q_\psi$$, $$b \in \mathbb {B}^{k+l}$$, $$\emptyset \ne M \subseteq \{\pi _1, \ldots , \pi _{k+l}\}$$, and $$A \subseteq \mathbb {B}^n$$;
- $$s_{ init }$$;
- $$(s_{ init }, A)$$ where $$A \subseteq \mathbb {B}^n$$.

As in Sect. 5, states $(\hat{s}, q, b)$ capture an abstract state $\hat{s} \in \mathbb {B}^n$, track the safety automaton $\mathcal {A}_\psi$, and record which copy has moved since the last update. States of this form are controlled by player $\texttt {SAFE}$ who, again, takes the role of the verifier. States $(\hat{s}, q, b, M, A)$ encode the scheduling *M* (as in Sect. 5) and also include a step-restriction *A*. These states are controlled by $\texttt {REACH}$, who, again, takes the role of the refuter. The step-restriction *A* enables the verifier (who will pick the restriction), to control which transition to take in existentially quantified copies (Sect. 6.4 gives details). State $s_ {init}$ serves as the initial state of the game. Different from the game in Sect. 5, player $\texttt {SAFE}$ controls $s_ {init}$. Lastly, states of the form $(s_{ init }, A)$ include an initial-restriction *A* (Sect. 6.4 gives details). Player $\texttt {REACH}$ controls states $(s_{ init }, A)$.

Formally, we define

5 $$\begin{aligned} \begin{aligned} S_\texttt {SAFE}:= \,&\big \{(\hat{s}, q, b) \mid \hat{s} \in \mathbb {B}^n \wedge q \in Q_\psi \wedge b \in \mathbb {B}^{k+l} \wedge \texttt {SAT}(\llbracket \hat{s} \rrbracket ) \big \} \, \cup \, \big \{s_ {init} \big \} \end{aligned} \end{aligned}$$

and

6 $$\begin{aligned} \begin{aligned} S_\texttt {REACH}:= \,&\big \{(\hat{s}, q, b, M, A) \mid \hat{s} \in \mathbb {B}^n \wedge q \in Q_\psi \wedge b \in \mathbb {B}^{k+l} \, \wedge \\&\quad \quad \quad \quad \emptyset \ne M \subseteq \{\pi _1, \ldots , \pi _{k+l}\} \wedge A \subseteq \mathbb {B}^n \wedge \texttt {SAT}(\llbracket \hat{s} \rrbracket ) \big \} \, \cup \\&\big \{ (s_ {init} , A) \mid A \subseteq \mathbb {B}^n \big \}. \end{aligned} \end{aligned}$$

As before we define $S:= S_\texttt {SAFE}\cup S_\texttt {REACH}$ as the set of all states.

#### Game transitions

**Fig. 6:**
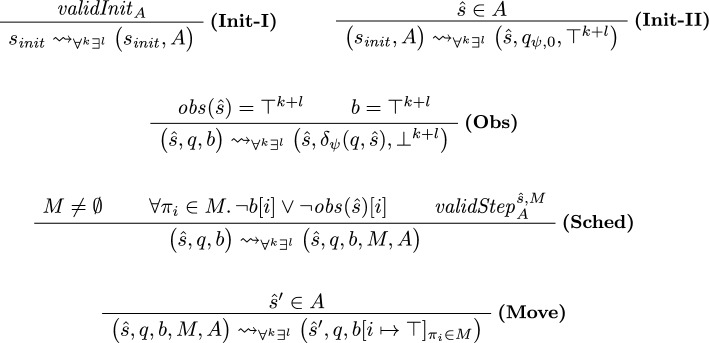
Transition rules for the verification of $\forall ^k\exists ^l$ properties

To reflect the effect of restrictions, we modify the transition rules of our game and depict the updated rules in Fig. 6.

***Picking an Initial Restriction (Init-I) and (Init-II).***

In the game from Sect. 5, the refuter controlls $s_ {init}$ and can pick *any* abstract initial state that contains some concrete initial state.^5^ In the $\forall ^k\exists ^l$ setting of this section we can be more precise. In the OHyperLTL semantics, we can pick some trace for all existentially quantified trace variables; in particular, in our game, the verifier (who controls existentially quantified traces) should be able to pick some initial state for all existentially quantified traces. On the level of abstract states, this means that the verifier can disallow certain abstract initial states. We reflect this in our rules:

In rule **(Init-I)**, the verifier (who controls $s_{ init }$) can choose a set of abstract states $A \subseteq \mathbb {B}^n$ under the condition that *A* constitutes a *valid initial restriction* (denoted $validInit _{A}$). Intuitively, $validInit _{A}$ asserts that the set of abstract states *A* is large enough such that, no matter which concrete initial states the refuter picks for universally quantified traces $\pi _1, \ldots , \pi _k$, the verifier can pick concrete initial states for $\pi _{k+1}, \ldots , \pi _{k+l}$ such that the combined initial state abstracts to an abstract state within *A*. We define $validInit _{A}$ formally in Sect. 6.3.

Afterward, using rule **(Init-II)**, the refuter (who controls state $(s_{ init }, A)$) can pick any abstract state $\hat{s} \in A$ and start the game from $(\hat{s}, q_{\psi , 0}, \top ^{k+l})$ (similar to the initial states in the the game from Sect. 5).

***Updating the Automaton State (Obs).***

Rule **(Obs)** stays as in Sect. 5, i.e., if all copies moved and are at an observation point, we update the automaton state and reset *b*.

***Picking a Scheduling and Restriction (Sched).***

In rule **(Sched)**, the safety player selects both a scheduling *M* and a set of abstract states $A \subseteq \mathbb {B}^n$ under the condition that *A* constitutes a *valid step restriction* (denoted $validStep ^{\hat{s},M}_{A}$). Intuitively, $validStep ^{\hat{s},M}_{A}$ asserts that the set of abstract states *A* is large enough such that the verifier (at the level of concrete states) can ensure a transition to an abstract state within *A*. We define $validStep ^{\hat{s},M}_{A}$ formally in Sect. 6.4.

***Moving the Copies (Move).***

Lastly, rule **(Move)** is applicable in states of the form $(\hat{s}, q, b, M, A)$ that already encode the scheduling and restriction. Here, the reachability player can pick any abstract state contained in the step restriction *A*.

***All Transitions.***

We define the transition relation as all state pairs that are derivable with the rules in Fig. 6, i.e.,

7 $$\begin{aligned} T := \big \{(\textbf{x}, \textbf{y}) \in S \times S \mid \textbf{x} \rightsquigarrow _{\forall ^k\exists ^l} \textbf{y} \big \}. \end{aligned}$$

#### Losing game states

Similar to the game constructed in Sect. 5, we mark a state $(\hat{s}, q, b)$ or $(\hat{s}, q, b, M, A)$ as losing iff the automaton state *q* is a bad state in $\mathcal {A}_\psi$, i.e.,

8 $$\begin{aligned} S_ {bad} := \big \{(\hat{s}, q, b) \in S \mid q \in B_\psi \big \} \cup \big \{(\hat{s}, q, b, M, A) \in S \mid q \in B_\psi \big \}. \end{aligned}$$

#### $\mathcal {G}^{\forall \exists }_{(\mathcal {T}, \varphi , \mathcal {P})}$

##### Definition 2

We define the safety game $\mathcal {G}^{\forall \exists }_{(\mathcal {T}, \varphi , \mathcal {P})}$ as

$$\begin{aligned} \mathcal {G}^{\forall \exists }_{(\mathcal {T}, \varphi , \mathcal {P})} := \big (S_\texttt {SAFE}, S_\texttt {REACH}, s_ {init} , T, S_ {bad} \big ) \end{aligned}$$

where $S_\texttt {SAFE}$, $S_\texttt {REACH}$, *T*, and $S_ {bad}$ are defined in (5), (6), (7), and (8), respectively.

### Valid initial restriction

In our game, the safety player can restrict the set of abstract initial states. Formally, we say a set of abstract states $A \subseteq \mathbb {B}^n$ is a *valid initial restriction*, written $validInit _{A}$, if the following formula holds

$$\begin{aligned}&\forall X_{\pi _1} \cup \cdots \cup X_{\pi _k}\mathpunct {.}\bigwedge _{i=1}^k init _{\langle \pi _i \rangle } \rightarrow \bigg (\exists X_{\pi _{k+1}} \cup \cdots \cup X_{\pi _{k+l}}\mathpunct {.}\bigwedge _{i=k+1}^{k+l} init _{\langle \pi _i \rangle } \wedge \bigvee _{\hat{s} \in A} \llbracket \hat{s} \rrbracket \bigg ). \end{aligned}$$

In this formula, we first quantify universally over concrete initial states for all *k* universally quantified variables, i.e., assignments to $X_{\pi _1} \cup \cdots \cup X_{\pi _{k}}$ that satisfies $init _{\langle \pi _i \rangle }$ for all $1 \le i \le k$. Afterward, we quantify existentially over concrete initial states for all *l* existentially quantified traces (assignments to $X_{\pi _{k+1}} \cup \cdots \cup X_{\pi _{k+l}}$) and assert that **(1)** the assignment constitutes valid initial states (i.e., satisfies $init _{\langle \pi _i \rangle }$ for all $k+1 \le i \le k+l$), and **(2)** the resulting assignment lies within an abstract states in *A*. Recall that $\llbracket \hat{s} \rrbracket$ is a formula over $\vec {X} = X_{\pi _1} \cup \cdots \cup X_{\pi _{k+l}}$.

Intuitively, this ensures that no matter what initial state the refuter picks for the *k* universally quantified copies, the verifier can pick initial states for the *l* existentially quantified such that an abstract state in *A* is reached. In particular, instead of reasoning about infinite traces (as in the OHyperLTL semantics), we only reason about the initial states of those traces in a local SMT query. A similar idea will be used in Sect. 6.4 to ensure that a step restriction is valid.

#### Example 4

Consider the STS $\mathcal {T}= (\{x\}, x \ge 5, x' = x)$ and the OHyperLTL property

$$\begin{aligned} \varphi = \forall \pi _1 : \top \mathpunct {.}\exists \pi _2 : \top \mathpunct {.}\Box (x_{\pi _1} = x_{\pi _2}) \end{aligned}$$

It is easy to see that $\mathcal {T}\models \varphi$. Now define $\mathcal {P}:= \{x_{\pi _1} = x_{\pi _2}\}$ and let $\hat{s}_1$ be the abstract state where $x_{\pi _1} = x_{\pi _2}$ holds and $\hat{s}_2$ be the abstract state where $x_{\pi _1} \ne x_{\pi _2}$. We can derive that $\{\hat{s}_1\}$ is a valid initial restriction as the following formula holds

$$\begin{aligned} validInit _{\{\hat{s}_1\}} = \,&\forall x_{\pi _1} \mathpunct {.}\underbrace{x_{\pi _1} \ge 5}_{ init _{\langle \pi _1 \rangle }} \rightarrow \Big (\exists x_{\pi _2} \mathpunct {.}\underbrace{x_{\pi _2} \ge 5}_{ init _{\langle \pi _2 \rangle }} \wedge \underbrace{x_{\pi _1} = x_{\pi _2} }_{\llbracket \hat{s}_1 \rrbracket }\Big ). \end{aligned}$$

In contrast, if we would start from the abstract states used in Sect. 5 (i.e., start from *all* abstract states that contain some combination of concrete initial states), we would mark both $\hat{s}_1$ and $\hat{s}_2$ as initial. In this case, $\texttt {SAFE}$ would not win as $\hat{s}_2$ already violates $\Box (x_{\pi _1} = x_{\pi _2})$.

### Valid step restriction

Similar to Sect. 6.3, we also use restrictions to restrict the set of abstract successor states in each step of the game. To this end, we approximate the $\forall ^k\exists ^l$ quantifier alternation in the OHyperLTL specification (which ranges over traces) by a local $\forall ^k\exists ^l$ first-order formula which only reasons about the behavior in a *single step*. Formally we define $validStep ^{\hat{s},M}_{A}$ as follows:

$$\begin{aligned}&\forall X_{\pi _1} \cup \cdots \cup X_{\pi _{k+l}}\mathpunct {.}\llbracket \hat{s} \rrbracket \rightarrow \\&\quad \quad \quad \Bigg (\forall X_{\pi _1}' \cup \cdots \cup X_{\pi _k}'\mathpunct {.}\bigwedge _{i=1}^k ite \big (\pi _i \in M, step _{\langle \pi _i \rangle }, \bigwedge _{x \in X} x_{\pi _i}' = x_{\pi _i}\big ) \rightarrow \\&\quad \quad \quad \quad \quad \quad \bigg (\exists X_{\pi _{k+1}}' \cup \cdots \cup X_{\pi _{k+l}}'\mathpunct {.}\\&\quad \quad \quad \quad \quad \quad \quad \quad \quad \bigwedge _{i=k+1}^{k+l} ite \Big (\pi _i \in M, step _{\langle \pi _i \rangle }, \bigwedge _{x \in X} x_{\pi _i}' = x_{\pi _i}\Big ) \wedge \bigvee \limits _{\hat{s}' \in A} \llbracket \hat{s}' \rrbracket ^{\langle '\rangle }\bigg )\Bigg ). \end{aligned}$$

In this formula, we first quantify universally over concrete states in $\hat{s}$, i.e., assignments to $X_{\pi _1} \cup \cdots \cup X_{\pi _{k+l}}$ that satisfy $\llbracket \hat{s} \rrbracket$. Afterward, we quantify (again universally) over concrete successor states for all universally quantified copies, i.e., assignments $X_{\pi _1}' \cup \cdots \cup X_{\pi _{k}}'$. Here, we only consider those steps that only move copies that are actually scheduled in *M*, i.e., for all $1 \le i \le k$ with $\pi _i \in M$ we require that $step _{\langle \pi _i \rangle }$ holds (which is a formula over $X_{\pi _i} \cup X_{\pi _i}'$) and for all $1 \le i \le k$ with $\pi _i \not \in M$ the assignment to $X_{\pi _i}'$ should agree with $X_{\pi _i}$ (the copy does not move). After we have fixed successor states for universally quantified copies, we quantify over concrete successor states for existentially quantified copies, i.e., assignments to $X_{\pi _{k+1}}' \cup \cdots \cup X_{\pi _{k+l}}'$. We require that **(1)** these successor states correspond to actual transitions in the system under *M*, and **(2)** the resulting next state abstracts to a state in *A*. Recall that $\llbracket \hat{s}' \rrbracket$ is a formula over $\vec {X} = X_{\pi _1} \cup \cdots \cup X_{\pi _{k+l}}$ so $\llbracket \hat{s}' \rrbracket ^{\langle ' \rangle }$ is a formula over $\vec {X}' = X_{\pi _1}' \cup \cdots \cup X_{\pi _{k+l}}'$.

#### Example 5

With this definition at hand, we can validate the step restrictions chosen by the strategy in Fig. 3c. In state $\alpha _5$ the strategy schedules $M = \{\pi _2\}$ and restricts the successor states to $\{\alpha _6\}$ even though abstract state
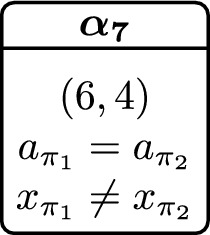


(which is not listed in Fig. 3c) is also a $\{\pi _2\}$-successor of $\alpha _5$. If we spell out $validStep ^{\alpha _5,\{\pi _2\}}_{\{\alpha _6\}}$ we get

$$\begin{aligned}&\forall x_{\pi _1}, a_{\pi _1}, x_{\pi _2}, a_{\pi _2}.\; \overbrace{a_{\pi _1} = a_{\pi _2}}^{\llbracket \alpha _5 \rrbracket } \rightarrow \\&\quad \quad \forall x_{\pi _1}', a_{\pi _1}'.\; (x_{\pi _1}' = x_{\pi _1} \wedge a_{\pi _1}' = a_{\pi _1} )\rightarrow \\&\quad \quad \quad \quad \exists x_{\pi _2}', a_{\pi _2}'\mathpunct {.}\\&\quad \quad \quad \quad \quad \quad \underbrace{a_{\pi _2}' = a_{\pi _2}}_{ step_{\langle {\pi _2} \rangle } } \wedge \underbrace{\big (a_{\pi _1}' = a_{\pi _2}' \wedge x_{\pi _1}' = x_{\pi _2}'\big )}_{\llbracket \alpha _6 \rrbracket ^{\langle '\rangle }} \end{aligned}$$

which holds. Here we assume that $step :=(a' = a)$ is the update performed on instruction $x \leftarrow \star _\mathbb {N}$ from Q2:3 to Q2:4 (which is slightly simplified compared to the actual transition formula, cf. Example 1).

### Soundness

We can show that if $\texttt {SAFE}$ wins $\mathcal {G}^{\forall \exists }_{(\mathcal {T}, \varphi , \mathcal {P})}$, the $\forall ^k\exists ^l$ property $\varphi$ holds on $\mathcal {T}$.

#### Theorem 3

If player $\texttt {SAFE}$ wins $\mathcal {G}^{\forall \exists }_{(\mathcal {T}, \varphi , \mathcal {P})}$, then $\mathcal {T} \models \varphi$.

#### Proof

Let $\sigma$ be a winning strategy for $\texttt {SAFE}$ in $\mathcal {G}^{\forall \exists }_{(\mathcal {T}, \varphi , \mathcal {P})}$. Let $t_1, \ldots , t_k\in Traces (\mathcal {T})$ be arbitrary. We use $\sigma$ to incrementally construct witness traces $t_{k+1}, \ldots , t_{k+l}$ by querying $\sigma$. In every abstract state $\hat{s}$, $\sigma$ selects a scheduling *M* and a restriction *A* such that $validStep ^{\hat{s},M}_{A}$ holds. We plug the current *concrete* state (reached in our construction of $t_{k+1}, \ldots , t_{k+l}$) into the universal quantification of $validStep ^{\hat{s},M}_{A}$ and get (concrete) witnesses for the existential quantification which, by definition of $validStep ^{\hat{s},M}_{A}$, are valid successors for the existentially quantified copies in $\mathcal {T}$. $\square$

#### Remark 3

Recall that we assume that for every existential quantification $\exists \pi _i: \xi _i$ occurring in $\varphi$ and all $t \in Traces (\mathcal {T})$, $valid (t,\xi _i)$ holds. This is important to ensure that the safety player (the verifier) cannot avoid observation points forever. We could drop this assumption by strengthening the winning condition in $\mathcal {G}^{\forall \exists }_{(\mathcal {T}, \varphi , \mathcal {P})}$ and explicitly state that, in order to win, $\texttt {SAFE}$ needs to visit observation points on existentially quantified traces infinitely many times.

***Clairvoyance vs. Abstraction.***

In general, the game-based method is incomplete, as the verifier cannot see the future of universally quantified traces [17]. The cooperation between reduction (the ability of the verifier to select schedulings) and witness strategy (the ability to select restrictions on the successor) can be seen as a limited form of prophecy [1, 17]. By first scheduling the universal copies, the witness strategy can peek at future moves before committing to a successor state, as we, e.g. saw in Sect. 2.2. The “theoretically optimal” reduction is thus a sequential one that first schedules only the universally quantified traces (until an observation point is reached) and thereby provides maximal information for the witness strategy. In the context of a fixed set of predicates, this reduction is, however, not always optimal as the gained information may not be captured within the abstraction. Our verification framework, therefore, strikes a delicate balance between the clairvoyance needed by the witness strategy and the precision captured in the abstraction, further emphasizing why the searches for reduction and witness strategy need to be mutually dependent.

However, even when extended with the ability to peek at future steps using reductions, incompleteness remains. Intuitively, our game limits the reduction to take effect between two observation points (to ensure that all traces synchronize at the observation points). Even the “theoretically optimal” reduction that schedules only universally quantified traces, can, therefore, only peek at the future up to the next observation point.

#### Example 6

Let $\mathcal {T}$ be a STS that can, in each step, non-deterministically pick a value of *x*. Now consider the property:

$$\begin{aligned} \forall \, \pi _1 : \top \mathpunct {.}\exists \, \pi _2 : \top \mathpunct {.}(x_{\pi _2} \ge 5) \leftrightarrow \bigcirc (x_{\pi _1} \ge 5) \end{aligned}$$

That is, for every trace $\pi _1$, there exists some trace $\pi _2$, such that the first value on $\pi _2$ is at least 5 iff the second value of $\pi _1$ is at least 5. The property clearly holds: In the OHyperLTL semantics, we first fix the *entire* trace $\pi _1$. When constructing a witnessing trace for $\pi _2$, we thus know if the second value on $\pi _1$ is at least 5 and can chose an appropriate value for *x* on $\pi _2$. However, we cannot verify the above property using our current game. As we observe every step ($\xi = \top$), the reduction effectively has to schedule in lock-step. The choice of *x* on $\pi _2$ in the first step, therefore, cannot peek at the value of *x* on $\pi _1$ chosen in the second step.

To counteract this incompleteness, we can employ prophecy variables [1] to predict future events on universally quantified traces (see, e.g., [17, 38]).

### $\mathcal {G}^{\forall \exists }_{(\mathcal {T}, \varphi , \mathcal {P})}$ generalizes $\mathcal {G}^\forall _{(\mathcal {T}, \varphi , \mathcal {P})}$

We can show that the idea of *restrictions* used in $\mathcal {G}^{\forall \exists }_{(\mathcal {T}, \varphi , \mathcal {P})}$ is closely related with the game we constructed in Sect. 5. In this subsection, we show **(1)** that the purely over-approximative ideas used in Sect. 5 are always valid restrictions in $\mathcal {G}^{\forall \exists }_{(\mathcal {T}, \varphi , \mathcal {P})}$ (in Sect. 6.6.1), and **(2)** that in the case of $\forall ^k$ properties, restrictions directly correspond to the concepts used in the *k*-safety game from Sect. 5 (in Sect. 6.6.2).

#### Restrictions by overapproximation

We can show that the purely over-approximative concepts used in Sect. 5 always yield valid restrictions.

***Valid Initial Restriction.***

In Sect. 5, we allow the refuter to start from any initial state that contains some initial state. Let us define

$$\begin{aligned} \textstyle A_{ maxInit } := \big \{ \hat{s} \in \mathbb {B}^n \mid \texttt {SAT}\big (\llbracket \hat{s} \rrbracket \wedge \bigwedge _{i=1}^{k+l} init _{\langle \pi _i \rangle }\big ) \big \} \end{aligned}$$

as the set of all those abstract states.

We can show that $A_{ maxInit }$ forms a valid initial restriction.

##### Lemma 4

$A_{ maxInit }$ is a valid initial restriction, i.e., $validInit _{A_{ maxInit }}$ holds.

On the other hand, as we saw in Example 4, the verifier can restrict the initial states to some proper subset of $A_{ maxInit }$.

***Valid Step Restriction.***

The game in Sect. 5 is based on the notation of an *M*-successor. Recall, that we write $\hat{s} \xrightarrow {M} \hat{s}'$ iff we can transition from some concrete state in $\hat{s}$ to some concrete state in $\hat{s}'$ by moving copies in $M \subseteq \{\pi _1, \ldots , \pi _{k+l}\}$. In particular, the abstract transition relation $\xrightarrow {M}$ is purely overapproximative, i.e., considers *all* possible transitions from concrete states. We define

$$\begin{aligned} Sucs (\hat{s}, M) := \big \{\hat{s}' \in \mathbb {B}^n \mid \hat{s} \xrightarrow {M} \hat{s}' \big \} = \big \{\hat{s}' \in \mathbb {B}^n \mid \texttt {SAT}\big ( \llbracket \hat{s} \rrbracket \wedge \llbracket \hat{s}' \rrbracket ^{\langle '\rangle } \wedge step _M \big )\big \} \end{aligned}$$

as the set of all *M*-successors of $\hat{s}$.

We can show that $Sucs (\hat{s}, M)$ always forms a valid step restriction.

##### Lemma 5

For any abstract state $\hat{s}$ and scheduling *M*, $Sucs (\hat{s}, M)$ is a valid step restriction, i.e., $validStep ^{\hat{s},M}_{ Sucs (\hat{s}, M) }$ holds.

On the other hand, as we saw in Example 5, a valid step restriction might be smaller (w.r.t. $\subseteq$) than $Sucs (\hat{s}, M)$.

***Minimal Restrictions.***

Lemmas 4 and 5 show that $A_{ maxInit }$ and $Sucs (M, \hat{s})$ are always valid restrictions. We can further show that any *minimal* valid initial (resp. step) restriction must be a subset of $A_{ maxInit }$ (resp. $Sucs (M, \hat{s})$).

##### Lemma 6

For any $A \subseteq \mathbb {B}^n$, we have $validInit _{A}$ iff $validInit _{A \cap A_{ maxInit }}$.

##### Lemma 7

For any abstract state $\hat{s}$, scheduling *M*, and restriction $A \subseteq \mathbb {B}^n$, we have $validStep ^{\hat{s},M}_{A}$ iff $validStep ^{\hat{s},M}_{A \cap Sucs (\hat{s}, M)}$.

While Lemmas 6 and 7 restrict the possible minimal valid restrictions, there does, in general, not exist a unique minimal restriction, as shown by the following example.

##### Example 7

Consider Example 5. From state $\alpha _5$ we get that $Sucs (\alpha _5, \{\pi _2\}) = \{\alpha _6, \alpha _7\}$ ($\alpha _7$ is depicted in Example 5). In Example 5, we have already shown that $\{\alpha _6\}$ is a valid step restriction. Analogously, we can show that $\{\alpha _7\}$ is also a valid restriction, i.e., the verifier can also enforce a transition to a state where the value of $x_{\pi _2}$ disagrees with that of $x_{\pi _1}$. Consequently, $\{\alpha _6\}$ and $\{\alpha _7\}$ are both valid step restrictions, and both are minimal w.r.t. $\subseteq$.

In $\mathcal {G}^{\forall \exists }_{(\mathcal {T}, \varphi , \mathcal {P})}$, we therefore include *all* valid restrictions and let the verifier pick an appropriate restriction.

#### Restrictions in the case of *k*-safety

Above, we saw that the over-approximate concepts used in Sect. 5 always yield valid restrictions. We can show that in the case of $\forall ^k$ OHyperLTL formulas, the over-approximation-based restrictions ($A_{ maxInit }$ and $Sucs (\hat{s}, M)$) are the unique minimal restrictions. This allows us to prove that $\mathcal {G}^{\forall \exists }_{(\mathcal {T}, \varphi , \mathcal {P})}$ *generalizes* the game $\mathcal {G}^\forall _{(\mathcal {T}, \varphi , \mathcal {P})}$ from Sect. 5 (when applied to $\forall ^k$ OHyperLTL formulas).

***Valid Initial Restrictions in the Case of*** *k*-***Safety.***

We already saw in Lemma 4 that $A_ {maxInit}$ always forms a valid initial restriction. If we consider valid initial restrictions in the case of $\forall ^k$-properties, the definition of $validInit _{A}$ simplifies to

$$\begin{aligned}&\forall X_{\pi _1} \cup \cdots \cup X_{\pi _k}\mathpunct {.}\bigwedge _{i=1}^k init _{\langle \pi _i \rangle } \rightarrow \bigvee _{\hat{s} \in A} \llbracket \hat{s} \rrbracket . \end{aligned}$$

In this special case, there exists a unique minimal valid initial restriction: $A_{ maxInit }$.

##### Lemma 8

Assume that $\varphi$ is a $\forall ^k$ OHyperLTL formula. For any set of abstract states $A \subseteq \mathbb {B}^n$, we have that $validInit _{A}$ holds if and only if $A_{ maxInit } \subseteq A$.

***Valid Step Restrictions in the Case of*** *k*-***Safety.***

The same reasoning also holds for valid step restrictions. If we consider $\forall ^k$ properties, the definition of $validStep ^{\hat{s},M}_{A}$ simplifies to

$$\begin{aligned}&\forall X_{\pi _1} \cup \cdots \cup X_{\pi _{k}}\mathpunct {.}\llbracket \hat{s} \rrbracket \rightarrow \Bigg (\forall X_{\pi _1}' \cup \cdots \cup X_{\pi _k}'\mathpunct {.}\\&\quad \quad \quad \bigg (\underbrace{\bigwedge _{i=1}^k ite \big (\pi _i \in M, step _{\langle \pi _i \rangle }, \bigwedge _{x \in X} x_{\pi _i}' = x_{\pi _i}\big )}_{ step _M} \bigg ) \rightarrow \bigvee \limits _{\hat{s}' \in A} \llbracket \hat{s}' \rrbracket ^{\langle '\rangle }\Bigg ). \end{aligned}$$

In this special case, there, again, exists a unique minimal valid step restriction for all abstract states $\hat{s}$ and schedulings *M*: $Sucs (\hat{s}, M)$.

##### Lemma 9

Assume that $\varphi$ is a $\forall ^k$ OHyperLTL formula. For every abstract state $\hat{s}$, scheduling *M*, and set of abstract states $A \subseteq \mathbb {B}^n$ we have that $validStep ^{\hat{s},M}_{A}$ holds if and only if $Sucs (\hat{s}, M) \subseteq A$.

***Unique Minimal Restrictions.***

In order to maximize the chance of winning, $\texttt {SAFE}$ wants to select restrictions that are as small as possible (w.r.t. $\subseteq$). Lemmas 8 and 9, therefore, tell us that—in the case of $\forall ^k$ properties—$A_{ maxInit }$ and $Sucs (\hat{s}, M)$ are the unique optimal restriction that $\texttt {SAFE}$ can pick. In Sect. 5, we, therefore, directly start in all abstract states within $A_{ maxInit }$ and always restrict the successors to $Sucs (\hat{s}, M)$. That is, we (implicitly) consider a unique restriction in each step. In particular, we can compute the sets $A_{ maxInit }$ and $Sucs (\hat{s}, M)$ locally by iterating over all abstract states; instead of checking all *sets of abstract states* for validity.

***Transfer of Winning Strategies.***

From Lemmas 8 and 9, it follows immediately that—in the case of $\forall ^k$ properties—the safety player cannot be more restrictive than selecting $A_{ maxInit }$ and $Sucs (\hat{s}, M)$ as restrictions. It follows that the winner of $\mathcal {G}^{\forall \exists }_{(\mathcal {T}, \varphi , \mathcal {P})}$ coincides with the winner of $\mathcal {G}^\forall _{(\mathcal {T}, \varphi , \mathcal {P})}$ in the case of $\forall ^k$ properties.

##### Proposition 10

Assume that $\varphi$ is a $\forall ^k$ OHyperLTL formula. Then $\mathcal {G}^{\forall \exists }_{(\mathcal {T}, \varphi , \mathcal {P})}$ is won by player $\texttt {SAFE}$ if and only if $\mathcal {G}^\forall _{(\mathcal {T}, \varphi , \mathcal {P})}$ (cf. Sect. 5) is won by player $\texttt {SAFE}$.

**Algorithm 1:**
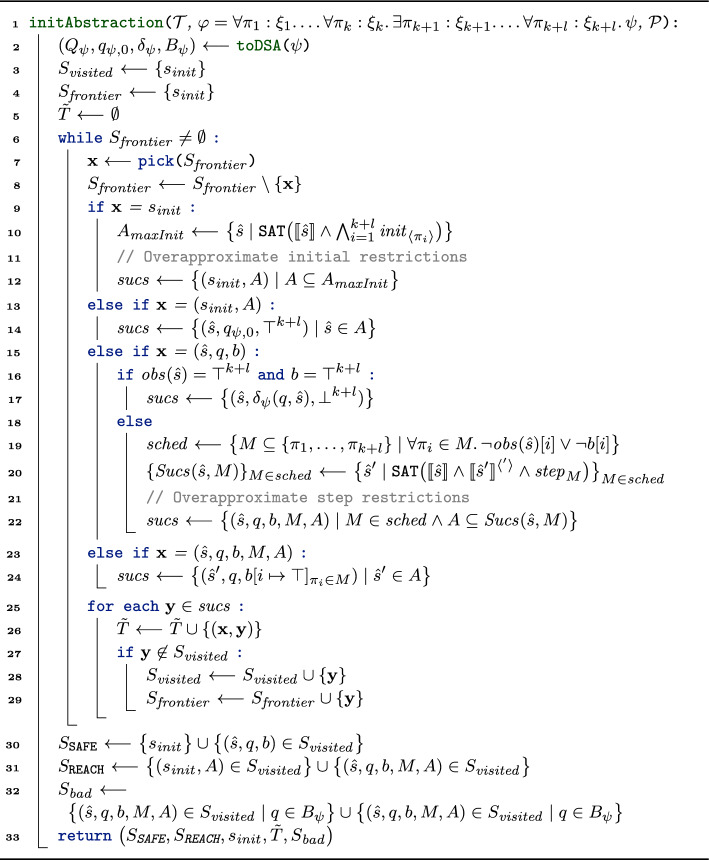
Algorithm for constructing the initial approximation of $\mathcal {G}^{\forall \exists }_{(\mathcal {T}, \varphi , \mathcal {P})}$. Function  converts an LTL formula to a deterministic safety automaton.

### Constructing and solving $\mathcal {G}^{\forall \exists }_{(\mathcal {T}, \varphi , \mathcal {P})}$

Constructing the game graph of $\mathcal {G}^{\forall \exists }_{(\mathcal {T}, \varphi , \mathcal {P})}$ in its entirety requires the identification of all valid restrictions (of which there are exponentially many in the number of abstract states and thus double exponentially many in the number of predicates). We propose a more effective algorithm that solves $\mathcal {G}^{\forall \exists }_{(\mathcal {T}, \varphi , \mathcal {P})}$ without (in the best case) constructing it explicitly. Instead, we operate on increasingly precise *approximations* of $\mathcal {G}^{\forall \exists }_{(\mathcal {T}, \varphi , \mathcal {P})}$. The idea is that each approximation allows the safety player to pick restrictions that might be *invalid*. We then eliminate such invalid restrictions lazily, i.e., we only check (and potentially eliminate) restrictions that are used by an actual strategy.

#### Constructing the initial overapproximation

Algorithm 1 constructs an initial overapproximation of $\mathcal {G}^{\forall \exists }_{(\mathcal {T}, \varphi , \mathcal {P})}$. The algorithm maintains a set $S_ {frontier}$ of all states that should be explored and a set $S_ {visited}$ that contains all states that have already been explored. In each iteration, we pick any game state $\textbf{x}$ in $S_ {frontier}$ (line 7) and remove it from $S_ {frontier}$. We then compute the set of all successor states $sucs$ of $\textbf{x}$ (lines 9 to 24) and add an edge from $\textbf{x}$ to all states in $sucs$ (line 26). However, instead of constructing the state space based on the actual transition rules of $\mathcal {G}^{\forall \exists }_{(\mathcal {T}, \varphi , \mathcal {P})}$ (cf. Fig. 6), Algorithm 1 approximates the set of restrictions. Concretely, in lines 12 and 22, we overapproximate the set of initial restrictions and step restrictions, as follows:

***Overapproximating Valid Initial Restrictions.***

According to rule **(Init-I)**, state $s_ {init}$ has edges to all states $(s_ {init} , A)$ where $validInit _{A}$ holds. As we saw in Lemma 6, any *minimal* valid initial restriction must be a subset of $A_{ maxInit }$. In line 10, we, therefore, first compute $A_{ maxInit }$, and in line 12, add an edge from $s_ {init}$ to all states $(s_ {init} , A)$ where $A \subseteq A_{ maxInit }$. This ensures that we add *all* minimal valid initial restrictions, but might include restrictions that are invalid. Importantly, we can compute $A_{ maxInit }$ locally, i.e., by iterating over abstract states opposed to *sets* of abstract states.

***Overapproximating Valid Step Restrictions.***

Using rule **(Sched) **(cf. Fig. 6), the safety player can, in a state $(\hat{s}, q, b)$, move to all states $(\hat{s}, q, b, M, A)$ where *M* is a valid scheduling and $validStep ^{\hat{s},M}_{A}$ holds. As for the initial states, we do not compute all valid step restrictions explicitly and instead overapproximate. In line 19, we compute all valid schedulings. For each valid scheduling *M*, we then include all step restrictions *A* where $A \subseteq Sucs (\hat{s}, M)$. As we saw in Lemma 7, any *minimal* valid step restriction is a subset of $Sucs (\hat{s}, M)$. We thus capture *all* minimal valid step restrictions but might include invalid restrictions.

#### Refining the overapproximation

The main algorithm for solving $\mathcal {G}^{\forall \exists }_{(\mathcal {T}, \varphi , \mathcal {P})}$ is depicted in Algorithm 2. It begins by computing an initial overapproximation of $\mathcal {G}^{\forall \exists }_{(\mathcal {T}, \varphi , \mathcal {P})}$ using Algorithm 1 and solves this approximation using a (standard) finite-state game solver (line 3). As argued in Sect. 6.7.1, this approximation might allow restrictions that are actually invalid and thus strengthens the power of the safety player. I.e., $\tilde{T}$ might include edges for $\texttt {SAFE}$ that are not part of $\mathcal {G}^{\forall \exists }_{(\mathcal {T}, \varphi , \mathcal {P})}$’s transitions. A winning strategy for $\texttt {REACH}$ in the overapproximation thus also works in $\mathcal {G}^{\forall \exists }_{(\mathcal {T}, \varphi , \mathcal {P})}$, but a winning strategy for $\texttt {SAFE}$ in the approximation might be invalid in $\mathcal {G}^{\forall \exists }_{(\mathcal {T}, \varphi , \mathcal {P})}$. To remedy this, we propose a simple refinement loop: whenever $\texttt {SAFE}$ wins the current approximation using some strategy $\sigma$, we check if all restrictions chosen by $\sigma$ are valid, i.e., we check validity lazily.

***Checking Initial Restrictions.***

In line 8, we query strategy $\sigma$ on state $s_ {init}$ to get the initial restriction *A* chosen by $\sigma$ and check if $validInit _{A}$ holds. If this restriction turns out to be invalid, we remove the corresponding transition $(s_ {init} , (s_ {init} , A))$ from $\tilde{T}$. Moreover, we do not only remove *A* but also all *subsets* of *A*; the set of all valid initial restrictions is upwards closed (w.r.t. $\subseteq$), so the set of invalid restrictions is downwards closed:

##### Lemma 11

Let $A \subseteq \mathbb {B}^n$ be any restriction with $\lnot validInit _{A}$. For any restriction $A'$ with $A' \subseteq A$, we have $\lnot validInit _{A'}$.

If we find that the initial restriction is invalid, we jump to line 3 and repeat.

**Algorithm 2:**
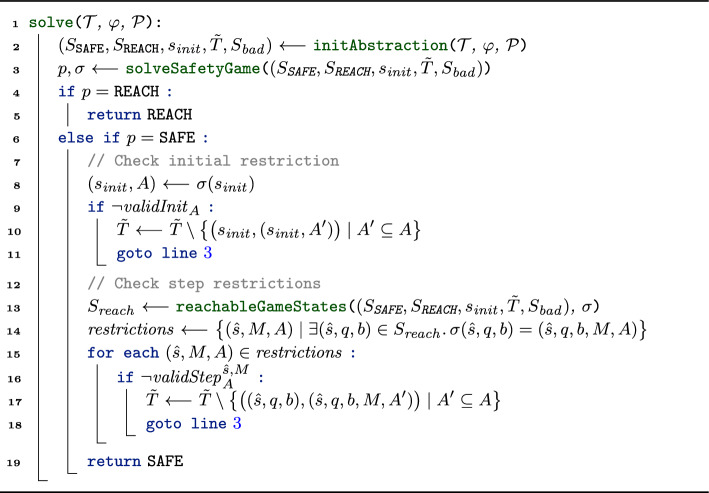
Iterative (lazy) solver for $\mathcal {G}^{\forall \exists }_{(\mathcal {T}, \varphi , \mathcal {P})}$. Function  solves a finite-state safety game and returns a pair $(p, \sigma )$ consisting of the winning player $p \in \{\texttt {SAFE}, \texttt {REACH}\}$ and a winning strategy $\sigma$ for player *p*. Function  computes all states that are reachable under a fixed strategy for $\texttt {SAFE}$.

***Checking Step Restrictions.***

Similarly, we check if all restrictions on transition steps are valid. For this, we first compute the set of all game states that are reachable under $\sigma$ (line 13). In a second step, we compute all step restrictions $(\hat{s}, M, A)$ that are chosen by $\sigma$ within this reachable fragment (line 14). If any restriction is invalid, we remove the corresponding transition from $\tilde{T}$ (line 17). Moreover—as for the initial states—we also remove all smaller restrictions as justified by the following lemma.

##### Lemma 12

Let $\hat{s} \in \mathbb {B}^n$ be any abstract state, *M* be any scheduling, and $A \subseteq \mathbb {B}^n$ be any restriction with $\lnot validStep ^{\hat{s},M}_{A}$. For any restriction $A'$ with $A' \subseteq A$, we have $\lnot validStep ^{\hat{s},M}_{A'}$.

If we find any invalid step restriction in the reachable fragment, we jump to line 3 and solve the (now smaller) game. If all initial and step restrictions are valid, we know that $\sigma$ is a winning strategy for $\texttt {SAFE}$ in $\mathcal {G}^{\forall \exists }_{(\mathcal {T}, \varphi , \mathcal {P})}$ and can thus return $\texttt {SAFE}$ as the winner (line 19).

***Searching for Maximal Restrictions.***

To improve the algorithm further, in line 3, we always compute a *maximal* safety strategy, i.e., a strategy for $\texttt {SAFE}$ that selects maximal restrictions (w.r.t. $\subseteq$). During the refinement, this allows us to eliminate many invalid restrictions from the overapproximation at the same time (cf. lines 10 and 17). For safety games, there always exists such a maximal winning strategy (see, e.g. [13]). Note that while each approximation is larger than $\mathcal {G}^{\forall \exists }_{(\mathcal {T}, \varphi , \mathcal {P})}$ (as we allow possibly invalid restrictions), solving this finite-state safety game can be done very efficiently (in linear time). The running time of Algorithm 2 is dominated by the SMT queries generated for each validity check. In practice, our refinement loop requires fewer validity checks than a full construction of $\mathcal {G}^{\forall \exists }_{(\mathcal {T}, \varphi , \mathcal {P})}$.

## Implementation and evaluation

**Table 1:** Evaluation of HyPA on $\forall ^k$ properties We give the size of the abstract game-space (Size), the time taken to compute the abstraction ($t_{abs}$), and the overall time taken by HyPA (*t*). Times are given in seconds

Instance	Size	${t}_{abs}$	*t*
DoubleSquareNI	819	92.3	92.8
HalfSquareNI	1166	85.9	86.5
SquaresSum	286	29.8	29.9
ArrayInsert	213	28.2	28.2
Exp1x3	112	4.5	4.5
Fig3	268	11.9	12.0
DoubleSquareNIff	121	9.8	9.9
Figure 2	333	23.7	23.8
ColIitem-Symm	494	24.0	24.1
Counter-Det	216	10.2	10.3
MultEquiv	757	18.9	19.0

When combining Theorem 3 and our iterative game solver (Algorithm 2) we obtain an algorithm to verify $\forall ^k\exists ^l$-safety properties within a given set of predicates. We have implemented a prototype of our method in a tool we call HyPA (short for **Hy**perproperty Verification with **P**redicate **A**bstraction). We use Z3 [48] to discharge SMT queries. The input of our tool consists of a *program graph* which features of a finite set of control locations and edges between locations are annotated with an arbitrary SMTLIB [5] formula over $X \cup X'$ describing the update performed on this edge. This allows us to, e.g., model the program counter directly within the control locations. Moreover, we can track predicates *locally* [62], i.e., for every $(k+l)$-tuple of control locations (representing the current control locations of all $k+l$ copies), we can use a distinct set of predicates.

### Evaluation on *k*-safety

As a special case of $\forall ^k\exists ^l$-safety properties, HyPA is also applicable to *k*-safety (i.e., $\forall ^k$) properties. We collected a small suite of programs and *k*-safety properties from various sources in the literature [54, 77–79, 79] and manually translated them into STSs (this can be automated easily). The results are given in Table 1. As done by Shemer et al. [77], we already provide a set of predicates that is sufficient for *some* reduction (but not necessarily the lockstep or sequential one), the search for which is then automated by HyPA. Our results show that the game-based search for a reduction can verify interesting *k*-safety properties from the literature. We also note that, currently, the vast majority of time is spent on the construction of the abstract system. If we would move to a fixed language, the computation time could be reduced by using existing (heavily optimized) tools for constructing a predicate abstraction [32, 62].

**Table 2:** Evaluation of HyPA on $\forall ^k\exists ^l$ properties. We give the size and construction time of the initial abstraction (Size and $t_{abs}$). For the direct and lazy (Algorithm 2) solver, we give the time to construct (and solve) the game ($t_{solve}$) and the overall time ($t = t_{abs} + t_{solve}$). For the lazy solver, we, additionally, give the number of refinement iterations (#Ref). Times are given in seconds and ‘–’ indicates a timeout after 5 min

			Direct		Lazy		
Instance	Size	${t}_{abs}$	${t}_{solve}$	*t*	#Ref	${t}_{solve}$	*t*
NonDetAdd	4568	3.5	–	–	4	1.0	4.5
CounterSum	479	5.3	9.1	14.4	17	0.9	6.2
AsynchGNI	437	6.1	6.9	13.0	1	0.1	6.2
CompilerOpt1	354	2.4	2.3	4.7	2	0.2	2.6
CompilerOpt2	338	2.8	2.4	5.2	2	0.2	3.0
Refine	1357	6.1	–	–	4	0.7	6.8
Refine2	1476	5.6	–	–	5	0.6	6.2
Smaller	327	2.3	4.0	6.3	11	0.4	2.7
CounterDiff	959	8.5	18.3	26.8	19	1.1	9.6
Figure 3	3180	11.1	–	–	22	2.9	14.0
P1 (simple)	83	2.0	1.4	3.4	1	0.1	2.1
P1 (GNI)	34793	17.0	–	–	72	95.7	112.7
P2 (GNI)	15753	10.2	–	–	7	5.1	15.3
P3 (GNI)	1429	6.6	20.9	27.5	7	0.6	7.2
P4 (GNI)	7505	16.5	–	–	72	13.2	29.7

### Evaluation on $\forall ^k\exists ^l$-safety

The main novelty of HyPA lies in its ability to, for the first time, verify temporal properties beyond *k*-safety. As none of the existing tools can verify such properties, we compiled a collection of very small example programs and $\forall ^k\exists ^l$-safety properties. Additionally, we modified the boolean programs from [16, 20] (where we checked GNI on boolean programs) by adding data from infinite domains.

Verifying the properties often requires a non-trivial combination of reduction and witness strategy (as the reduction must e.g., compensate for branches of different lengths). As before, we provide a set of predicates and let HyPA automatically search for a witness strategy with accompanying reduction. We list the results in Table 2. To highlight the effectiveness of our inner refinement loop, we apply **(1)** a *direct* solver that constructs $\mathcal {G}^{\forall \exists }_{(\mathcal {T}, \varphi , \mathcal {P})}$ in its entirety and then solves it, and **(2)** the lazy (iterative) solver given in Algorithm 2. Our lazy solver (Algorithm 2) clearly outperforms an explicit construction and is often the only method to solve the game in reasonable time. In particular, we require few refinement iterations and, therefore, also few expensive SMT validity queries. Unsurprisingly, the problem of verifying properties beyond *k*-safety becomes much more challenging (compared to *k*-safety verification) as it involves the *synthesis* of a witness function which is already 2-EXPTIME-hard for finite-state systems [17, 74].

## Related work

***Asynchronous Hyperproperties.***

Recently, many logics for the formal specification of asynchronous hyperproperties have been developed [10, 11, 16, 21, 30, 31, 59, 60]. Our logic OHyperLTL is closely related to stuttering HyperLTL (HyperLTL$_S$) [30]. In HyperLTL$_S$, each temporal operator is endowed with a set of temporal formulas $\Gamma$ and steps where the truth values of all formulas in $\Gamma$ remain unchanged are ignored during the operator’s evaluation. As for most mechanisms used to design asynchronous hyperlogics, finite-state model checking of HyperLTL$_S$ is undecidable. By contrast, in OHyperLTL, we always observe the trace at *fixed* locations (determined by the observation formula), which is key for ensuring decidable finite-state model checking.

*k*-***Safety Verification.***

The literature on *k*-safety verification is rich. Many approaches verify *k*-safety using a form of self-composition [8, 35, 51, 56] and often employ reductions to obtain compositions that are easier to verify. Our game-based interpretation of a reduction (Sect. 5) is closely related to Shemer et al. [77], who study *k*-safety verification using property-directed self-composition within a given predicate abstraction. Any winning strategy for $\texttt {SAFE}$ in our *k*-safety game corresponds to such a property-directed self-composition, and conversely, from any property-directed self-composition we can extract a winning strategy for $\texttt {SAFE}$. Shemer et al. [77] search for a reduction using an optimized enumeration of all reduction (by employing clever pruning strategies), whereas we phrase the search as a game. This game-based approach forms the key basis that allows our method to extend to hyperliveness properties. Farzan and Vandikas [54, 55] present a counterexample-guided refinement loop that simultaneously searches for a reduction and a proof. Different from our work, they consider trace abstractions [61] and automate the search for a reduction using a restricted class of tree automata. Eilers et al. [51] propose the notation of a modular product program as a representation of different compositions within the same program.

***Program Logics for*** *k*-***Safety.***

Along a different line of work, researchers have developed relational program logics [2, 9, 12, 50, 71, 78]. These logics reason about *k*-safety properties at the source-code level and thus benefit from the syntax-guided nature of the verification problem. In contrast, our approach is, in theory, applicable to all (infinite-state) systems that can be described as symbolic transition systems, including (but not limited to) programs.

$$\forall ^k\exists ^l$$-***Verification.***

Barthe et al. [7] describe an asymmetric product of the system such that only a subset of the behavior of the second system is preserved, thereby allowing the verification of $\forall ^k\exists ^l$ properties. Constructing an asymmetric product and verifying its correctness (i.e., showing that the product preserves all behavior of the first, universally quantified, system) is challenging. Unno et al. [79] present a constraint-based approach to verify functional (opposed to temporal) $\forall ^k\exists ^l$ properties in infinite-state systems using an extension of constraint Horn clauses called pfwCHC. The underlying verification approach is orthogonal to ours: pfwCHC allows for a clean separation of the actual verification and verification conditions, whereas our approach combines both. For example, our method can prove the existence of a witness strategy without ever formulating precise constraints on the strategy (which seems challenging). While most relational program logics focus on *k*-safety properties, recently logics for the verification of richer hyperproperties (including $\forall ^k\exists ^l$ properties) have been studied [14, 42, 44, 49]. Our work differs from these logics as we target *temporal* properties that reason about infinite executions. In finite-state systems, handling quantifier alternations in a hyperproperty specification is possible using automata-based techniques such as complementations [56], and inclusion checks [19, 23]. In infinite-state systems (the main motivation for this work), such techniques are not applicable. Pommellet and Touili [76] study the verification of HyperLTL in infinite-state systems arising from pushdown systems. By contrast, this work studies verification in infinite-state systems that arise from infinite variable domains, as, e.g., encountered in software. Other verification techniques for infinite-state systems rely on unrolling of the system using bounded model-checking [64] and symbolic execution [27, 41].

***Game-based Verification of Hyperliveness.***

Coenen et al. [38] introduce the game-based reading of existential quantification to verify temporal $\forall ^k\exists ^l$ properties in a synchronous and finite-state setting. By contrast, our work constitutes the first verification method for temporal $\forall ^k\exists ^l$-safety properties in *infinite-state* systems. The key to our method is a careful integration of reductions which is not possible in a synchronous setting. For finite-state systems (where the abstraction is precise) and synchronous specifications (where we observe every step), our method subsumes the one in [17, 26, 38]. In a finite-state setting, the game-based verification of $\forall ^k\exists ^l$ can be made complete by adding *prophecies* [17]. Automatically constructing prophecies for infinite-state systems is interesting future work. Recently, Correnson and Finkbeiner [40] use a coinductive interpretation of strategies, e.g., allowing the use in proof assistants.

***Beyond*** $$\forall ^k\exists ^l$$.

The game-based method used in our approach is, in its current form, limited to the verification of $\forall ^k\exists ^l$ properties. For $\forall ^k\exists ^l$ properties, the verifier (who constructs witness traces for existentially quantified trace variables) can observe the current state of the *k* universally quantified traces; similar to the OHyperLTL semantics where the choice for existentially quantified traces can depend on the traces chosen for earlier quantifiers. The information available to the verifier in our game (i.e., the abstraction of the prefixes of all traces) is thus a *subset* of the information available in the OHyperLTL semantics (where all universally quantified traces are fixed). If we move beyond $\forall ^k\exists ^l$ and include an $\exists \forall$ alternation the verifier can no longer base its choice on the current state of all system copies. For example in a $\exists \pi _1. \forall \pi _2$ property, the choice for $\pi _1$ must *not* depend on the current state of $\pi _2$. In finite-state systems, we can counteract this by considering a game played under partial information (see, e.g., [22, 24] for details). Studying if this idea can be extended to infinite-state systems—where we abstract the joint state space via predicates—is interesting future work.

***Encoding in CHC Satisfiability.***

In recent work, Itzhaky et al. [65] show that the game-based approach proposed in this paper is well suited for a reduction into constraint Horn clauses (CHC). Concretely, they show that a combination of reduction and witness strategy can be encoded into a CHC problem that views the reduction and witness strategy as unknown predicates.

***Predicate Abstraction and Underapproximation.***

(Predicate) abstraction-based verification methods have a long history in computer science. Traditionally, predicate abstractions *overapproximate* transitions in the system, i.e., include a transition from abstract states $\hat{s}_1$ to $\hat{s}_2$ iff some concrete state in $\hat{s}_1$ has a transition to some concrete state in $\hat{s}_2$ [58]. However, over the years, many approaches have combined predicate abstraction with *underapproximation* [72], perhaps most prominently when proving the *non*-termination of a program [34, 39, 67]. For example, Kuwahara et al. [67] explores path of a program and, in order to ensure an underapproximation, computes *sufficient* condition that can be taken by at least one branch. Our definition of valid restrictions (cf. Sect. 6.4) shares a similar idea, but explores it in the setting of $\forall ^k\exists ^l$ properties.

***Game Solving.***

Our game-based interpretations are naturally related to infinite-state game solving [4, 28, 52, 53, 80]. State-of-the-art solvers for infinite-state games unroll the game [53], use necessary subgoals to inductively split a game into subgames [4], encode the game as a constraint system [28], iteratively refine the controllable predecessor operator [80], and encode finite choices as constraints Horn clauses [52]. We tried to encode our verification approach directly as an infinite-state linear-arithmetic game. However, existing solvers (which, notably, work *without* a user-provided set of predicates) could not solve the resulting game [4, 53]. Our method for encoding the witness strategy using *restrictions* corresponds to hyper-must edges in general abstract games [46, 47]. Our inner refinement loop for solving a game with hyper-must edges without explicitly identifying all edges (Algorithm 2) is thus also applicable in general abstract games.

## Conclusion

In this work, we have presented the first verification method that can verify temporal hyperproperties with quantifier alternations in infinite-state systems. Our method is based on a game-based interpretation of reductions and existential trace quantification and allows for mutual dependence of both.

***Future Work.***

Currently, our method works within a fixed set of predicates (which we assume to be provided by the user). In the future, it is interesting to integrate our method in a counter-example guided refinement loop that automatically refines the abstraction. Moreover, one can attempt to lift the current restriction to temporally safe specification, i.e., tackle properties of the form $\forall ^k\exists ^l\mathpunct {.}\psi$ where $\psi$ is an arbitrary (not necessarily safety) property. Here, existing techniques for the verification of liveness properties using predicate abstraction might prove helpful [75]. More generally, it is interesting to study if, and to what extent, the numerous other methods developed for *k*-safety verification of infinite-state systems (including, e.g., trace abstractions and programs logics) are applicable to the vast landscape of hyperproperties that lies beyond *k*-safety.

## Data Availability

not applicable

## Footnotes

1 HyperLTL [36] can—similar to LTL [73]—access traces via their atomic propositions, which we can think of as Boolean variables. This limits HyperLTL’s expressiveness in infinite-state system where variables usually range over non-Boolean (potentially infinite) domains such as integers. In the variant of HyperLTL we consider here, we can refer to the (potentially non-Boolean) values of variables on traces. For example, $l_{\pi _1}$ refers to the value of variable *l* in the current time step on trace $\pi _1$. The atomic formula $l_{\pi _1} = l_{\pi _2}$ then states that (at the current timepoint) the value of *l* is equal on traces $\pi _1$ and $\pi _2$. For more details, see our temporal logic OHyperLTL defined in Sect. 4.

2 The term *hyperliveness* stems from the fact that—due to the existential quantification—GNI reasons about the existence of a particular execution. Similar to the definition of liveness in temporal properties [3], any set of execution traces can therefore satisfy GNI by *adding* sufficiently many traces.

3 The LTL specification is translated to a symbolic safety automaton that moves alongside the game. For the sake of readability, we omitted the automaton from the following discussion.

4 Note that in $\alpha _8$, the predicate $y_{\pi _1} = 2y_{\pi _2} + 1$ implies that $y_{\pi _1} > 0$ (as both variables range over $\mathbb {N}$), so when scheduling $M = \{\pi _1\}$, P1 is guaranteed to re-enter the inner loop.

5 In Sect. 5, all traces are universally quantified, so we need to establish the property from all possible combinations of initial states.
